# Tumoral periprostatic adipose tissue exovesicles-derived miR-20a-5p regulates prostate cancer cell proliferation and inflammation through the *RORA* gene

**DOI:** 10.1186/s12967-024-05458-3

**Published:** 2024-07-15

**Authors:** Silvia Sánchez-Martin, Antonio Altuna-Coy, Verónica Arreaza-Gil, Xana Bernal-Escoté, Joan Francesc Garcia Fontgivell, Helena Ascaso-Til, José Segarra-Tomás, Xavier Ruiz-Plazas, Matilde R. Chacón

**Affiliations:** 1 Disease Biomarkers and Molecular Mechanisms Group. IISPV. Joan, XXIII University Hospital, Universitat Rovira i Virgili, Tarragona, Spain; 2 Urology Unit, Joan XXIII University Hospital, Tarragona, Spain; 3 Pathology Unit, Joan XXIII University Hospital, Tarragona, Spain; 4 Institut d’Investigació Sanitària Pere Virgili. Hospital Universitari de Tarragona Joan XXIII, C/ Dr. Mallafré Guasch, 4, Tarragona, 43007 Spain

**Keywords:** Periprostatic adipose tissue, Exovesicles, miRNAs, Prostate cancer, *RORA* gene

## Abstract

**Background:**

From the first steps of prostate cancer (PCa) initiation, tumours are in contact with the most-proximal adipose tissue called periprostatic adipose tissue (PPAT). Extracellular vesicles are important carriers of non-coding RNA such as miRNAs that are crucial for cellular communication. The secretion of extracellular vesicles by PPAT may play a key role in the interactions between adipocytes and tumour. Analysing the PPAT exovesicles (EVs) derived-miRNA content can be of great relevance for understanding tumour progression and aggressiveness.

**Methods:**

A total of 24 samples of human PPAT and 17 samples of perivesical adipose tissue (PVAT) were used. EVs were characterized by western blot and transmission electron microscopy (TEM), and uptake by PCa cells was verified by confocal microscopy. PPAT and PVAT explants were cultured overnight, EVs were isolated, and miRNA content expression profile was analysed. Pathway and functional enrichment analyses were performed seeking potential miRNA targets. In vitro functional studies were evaluated using PCa cells lines, miRNA inhibitors and target gene silencers.

**Results:**

Western blot and TEM revealed the characteristics of EVs derived from PPAT (PPAT-EVs) samples. The EVs were up taken and found in the cytoplasm of PCa cells. Nine miRNAs were differentially expressed between PPAT and PVAT samples. The *RORA* gene (RAR Related Orphan Receptor A) was identified as a common target of 9 miRNA-regulated pathways. In vitro functional analysis revealed that the *RORA* gene was regulated by PPAT-EVs-derived miRNAs and was found to be implicated in cell proliferation and inflammation.

**Conclusion:**

Tumour periprostatic adipose tissue is linked to PCa tumour aggressiveness and could be envisaged for new therapeutic strategies.

**Supplementary Information:**

The online version contains supplementary material available at 10.1186/s12967-024-05458-3.

## Background

Prostate cancer (PCa) is the second leading cause of cancer-related death in most Western countries [1]. Its incidence has increased markedly since the 1990s due to the use of the prostate-specific antigen (PSA) test, eating habits and, aging [2]. PCa often develops slowly and initially remains confined to the prostate, causing minimal harm. However, aggressive forms can spread to bones and lymph nodes, leading to significant morbidity and mortality [3]. A central challenge in the management of PCa is discriminating between indolent and aggressive disease. Thus, early detection of PCa is important to guide treatment strategies [4]. Research in this area aims to enhance patient outcomes through a better understanding of the molecular mechanisms underlying PCa progression.

The biology of solid tumours should be analysed considering the tumour microenvironment (TME) [5]. TME is composed of stromal cells, including fibroblasts, immune cells, endothelial cells, and extracellular matrix cells [5]. However, since many cancers develop in the vicinity of adipose tissue (AT), peritumoral adipose tissue, and its associated adipocytes have already been reported to play a role in tumour initiation, progression, and drug resistance [6].

Periprostatic adipose tissue (PPAT) is the fatty tissue surrounding the prostate gland. The thickness of this fat depot, measured by magnetic resonance [7] or ultrasonography [8] was initially proposed as an aggressiveness marker for PCa. The first molecular indications that PPAT could condition the progression of PCa came from in vitro experiments using media from ex vivo PPAT cultures applied to PCa cell lines. In these experiments, changes in cell migration behaviour were observed [9], and molecules such as chemokine CCL7 (C-C Motif Chemokine Ligand 7) secreted by PPAT-adipocytes were demonstrated to stimulate the migration of tumour cells expressing CCR3 (CCL7 chemokine receptor) [10]. Other molecules such as IL-6 (Interleukin 6), Leptin [11], MMP-9 (Matrix Metallopeptidase 9) [9] and TGFα (Transforming Growth Factor alpha) [12] have also been reported to be highly expressed by PPAT and implicated in PCa progression [13]. Regarding PCa treatment, it has been demonstrated that PPAT can affect the response to DCTX (docetaxel) treatment by upregulating the expression of BCL-Xl (B‐cell lymphoma extra-large), BCL‐2 (B‐cell lymphoma 2), and TUBB2B (β‐tubulin isoform 2B). AG1024, a well‐known IGF‐1 (Insulin Like Growth Factor 1) receptor inhibitor, counteracts the decreased response to DCTX [14].

In addition, PCa cells have been shown to uptake metabolites secreted by PPAT, such as fatty acids, and used them as an energy source [15]. Ex vivo co-culture experiments using explants of PPAT and PCa cells reinforced the role of PPAT in aggravating tumour aggressiveness, as the expression of adhesion and proliferative-related genes (MMP-9 and TWIST1 (Twist Family BHLH Transcription Factor 1), lipid uptake, and lipid accumulation were increased in co-cultured PCa cells [15].

PPAT can communicate with the TME through EVs which are considered to play an important role in cell-to-cell communication. These EVs are broadly classified into apoptotic bodies, exosomes, and microvesicles [16]. Although the average size of EVs subtypes is different, their size range overlaps, and current EV-isolation methods do not allow accurate separation of the EV subtypes. Therefore the operative terms for EV subtypes recommended by the International Society for Extracellular Vesicles (ISEV) [16] refer to a physical characteristic of EVs, such as size like: “small EVs” < 100 nm or < 200 nm and “medium/large EVs” > 200 nm [17].

EVs facilitate the transfer of bioactive molecules: proteins, lipids, and nucleic acids, including miRNAs, a small non-coding RNA molecule that can regulate gene expression at the post-transcriptional level through degradation or repression and shows long-term stability in circulation [18].

Peritumoral adipose tissue derived-EVs have been demonstrated to modulate the acquisition and maintenance of cancer hallmark traits in melanoma [19] or breast cancer [20]. For instance, preadipocyte-secreted EVs that contain miR-140 have been shown to enhance breast tumorigenesis by regulating differentiation, and migration [21]. In ovarian cancer, miR-124-3p mesenchymal stem cell-derived EVs is a critical factor for inducing anti-proliferation signalling [22].

Thus, given that the presence of PPAT can favour tumour aggressiveness by mechanisms not yet fully characterized, we performed a human PPAT-derived EVs (PPAT-EVs) analysis concerning miRNA-cargo composition because this information may provide an opportunity to understand PCa progression better and may help to identify new molecular targets.

## Methods

### Patient recruitment and adipose tissue collection

Fresh AT samples were obtained from *n* = 25 patients laparoscopically assisted with a *Da Vinci* robot surgery at the Joan XXIII University Hospital of Tarragona (Spain). Once the anterior surface of the prostate was surgically exposed, 1–2 g of the surrounding fat tissue (PPAT) from *n* = 24 patients was removed for further processing. A non-tumorous extraperitoneal AT sample (1–2 g) (PVAT) was also removed during surgery from *n* = 17 patients. Fat samples were immediately washed twice in 1x PBS and used for in vitro explant culture experiments. Written informed consent before their inclusion in the study was provided by all patients. The study was approved by our local ethics committee and conducted following the provisions of the Declaration of Helsinki (Biomedical Research Law 14/2007, Royal Decree of Biobanks 1716/2011, Organic Law 15/1999 of September 13 Protection of Personal Data). Patients were stratified based on the International Society of Urological Pathology (ISUP) consensus conference on Gleason grading of prostatic carcinoma [23] as low-risk (ISUP I and II) and high-risk (ISUP III, IV, and V). Clinical parameters, tumour aggressiveness, and metabolic status of all patients were documented (Additional File 1: Table S1 and S2). All methods were approved and performed according to the guidelines and regulations of the Ethical Committee for Clinical Research (CEIm) from Pere Virgili Research Institute (CEIM205/2020). The inclusion criteria for patients were as follows: older than 18 years, diagnosed with PCa by prostate biopsy at our centre or any other centre, and treated by radical prostatectomy at our centre. Exclusion criteria were patients with a previous history of cancer, patients older than 75 years, and those who had received any previous treatment before radical prostatectomy for PCa.

### Adipose tissue explant culture

PPAT and PVAT explants were washed twice with 1x PBS supplemented with 1x antibiotic-antimycotic solution (Gibco, Fisher Scientific, Madrid, Spain) and 5 µg/mL Plasmocin (Invivogen, IBIAN Technologies, Zaragoza, Spain). Then, samples were centrifuged (280×g, 2 min, 22ºC) to eliminate any remaining blood cells. Approximately 1–2 g of PPAT or PVAT explants were dissected into pieces and plated in 12 well-plates (~ 4–6 pieces of 4 mm per well) with M199 medium supplemented with Foetal Bovine Serum (FBS) EVs-depleted (Gibco, Fischer Scientific S.L., Madrid, Spain), 25 mM HEPES (Gibco, Fischer Scientific S.L., Madrid, Spain), 1x antibiotic-antimycotic solution (Gibco, Fischer Scientific S.L., Madrid, Spain) and 5 µg/mL Plasmocin (Invivogen, Zaragoza, Spain). Explants were cultured in a humidified 5% CO_2_ atmosphere at 37 °C for 24 h. Conditioned culture media was then collected, filtered to exclude particles larger than 0.8 μm (Sartorius Minisart™ NML, Fischer Scientific S.L., Madrid, Spain), and frozen at -80ºC until EVs were isolated.

### Transmission electron microscopy (TEM)

Isolated EVs were placed on carbon-coated copper grids (200 mesh) and incubated in osmium tetroxide vapor for 15 min. Images were collected using a JEOL 1011 transmission electron microscope (Jeol, Tokyo, Japan) operating at 80 kV with a Megaview III camera (Olympus Soft Imaging Solutions GmbH, Munster, Germany).

### EVs uptake

PCa cells were seeded overnight in an 8-well Millicell^®^ EZ Chamber Slide (Sigma-Aldrich, Barcelona, Spain) at a density of 40.000 cells/cm^2^. Subsequently, cells were depleted overnight. The PPAT-EVs were labelled with PKH67 Green Fluorescent Cell Linker Kit (Sigma Aldrich, Saint Louis, MO, USA) as indicated by the manufacturer’s protocol. Cells were incubated with 20 µg/ml of PKH-67-labeled EVs at 37 °C for 1 h in a humidified 5% CO_2_ atmosphere. Conditioned M199 medium with FBS EVs-depleted was used as a negative control. After EVs treatment, cells were washed twice with 1x PBS and fixed in 3,7% (w/v) Paraformaldehyde for 1 h at room temperature. Fixed cells were washed three times with ice-cold PBS and permeabilized with 0,1% Triton X-100 for 10 min at room temperature. Then, cells were washed again three times with ice-cold 1x PBS and mounted using a coverslip with DAPI (Ibidi Mounting Medium, Planegg, Germany) to stain the cell nucleus. The images were recorded on the Leica TCS SP5 laser scanning spectral confocal microscope (Leica Microsystems Heidelberg) and further processed by FIJI (http://fiji.sc/) and Photoshop software.

### Extraction of EVs-derived miRNAs from adipose tissue explants and qRT-PCR profiling

ExoRNeasy Serum/Plasma Maxi Kit (Qiagen, Bionova, Barcelona, Spain) was used to isolate EVs from 16 mL of explant culture media from 4 ISUP high-risk patients matched for age: 4 PPAT samples and their paired PVAT samples. Subsequently, miRNAs from the obtained EVs were extracted using the ExoRNeasy Serum/Plasma Maxi Kit (Part II: Isolation of RNA) (Qiagen, Bionova, Barcelona, Spain). miRCURY LNA Universal RT microRNA PCR, Polyadenylation, and cDNA Synthesis Kit (Qiagen, Bionova, Barcelona, Spain) was used for reverse transcription. The miRNA profile contained in EVs was characterized by Quantitive Real Time Polymerase chain reaction (qRT-PCR) using ExiLENT SYBR Green Master Mix in the miRCURY LNA miRNA miRNome PCR Panel, Human Panel I + II, V5 (Qiagen, Bionova, Barcelona, Spain) that includes 752 human cancer-related mature miRNAs, according to the user’s protocol on a 7900HT Fast qRT-PCR System (Applied Biosystems, Foster City, CA, USA). Fluorescence readings and miRNA expression recordings were performed using SDS 2.3 software (Applied Biosystems, Foster City, CA, USA) and raw microarray data were extracted by Design and Analysis Software v.2.6.0 (DA2) (Applied Biosystems). Analysis of raw microarray qRT-PCR data was performed by Geneglobe Data Analysis Software (https://geneglobe.qiagen.com/us/analyze). The data was normalised using UniSp3 miRNA values to eliminate inter-microarray plate differences. A cycle threshold (C_T_) cut-off of < 35 was applied. C_T_ values for each sample were normalized to the arithmetic mean of 4 miRNAs (hsa-miR-423-5p, hsa-miR-103a-3p, hsa-miR-191-5p, and hsa-miR-16-5p) that showed no differences between studied groups [24]. The resulting value is known as ΔC_T_ sample. A calibrator (a sample made by mixing several AT samples) was included for the comparison of the different groups. Thus, each miRNA, regardless of the condition, was normalized to the ΔC_T_ of the calibrator sample (ΔΔC_T_ = ΔC_T_ sample -ΔC_T_ calibrator). The fold change expression of each miRNA was calculated with the formula 2^−ΔΔCT^. miRNAs with *p-value* ≤ 0.05 and C_T_ < 35 and, expression values ≥ 1.8-fold or ≤ -1.8-fold were considered for further validation analysis. Selected miRNAs were further validated in *n* = 24 samples of low-risk (ISUP Group I and II) and high-risk (ISUP Group III, IV, and V) PPAT and *n* = 17 PVAT samples.

### *In silico* EVs-derived miRNAs target analysis, pathway, and functional enrichment prediction

miRNet (https://www.mirnet.ca*)* was used to predict miRNA targets. Potentially altered pathways related to the targets were analysed using the Reactome database (https://reactome.org*).* The STRING database (https://string-db.org*)* was used to predict protein-protein interaction networks and to perform functional enrichment analysis. The STarMir web service (publicly available at: https://sfold.wadsworth.org/cgi-bin/starmirb.pl) was used to predict miRNA binding sites on selected target genes in the 3’UTR-seed region using the human model based cross-linked immunoprecipitation prediction model. For each of the miRNA-seed sites, STarMir provides the logistic probability of miRNA: hybrid target prediction, thus, miRNAs with > 1 interactions were considered for further analysis [25].

### *In silico* evaluation of *RORA* and selected miRNAs expression

The expression of *RORA* gene and the expression of selected miRNAs were evaluated in 52 non-pathogenic prostate tissue (NPP) and human prostate tumour tissue (PTT) using the data collected from the Cancer Genome Atlas Prostate Adenocarcinoma Prostate Cancer Database (TCGA-PRAD) supported by the CancerMIRNome database (publicly available at: http://bioinfo.jialab-ucr.org/CancerMIRNome/) (Additional File 1: Table S3). CancerMIRNome database, enables interactive analysis and visualization of miRNA expression profiles based on 33 cancer types from the TCGA, making it a useful tool to identify novel dysregulated miRNAs for cancer diagnosis or prognosis. Clinical data from samples was also downloaded from the TCGA-PRAD Data Portal.

### Paraffined PCa tissue RNA extraction

RNA was extracted from 6 slices of 5 µM/slice (1 cm^2^) formalin-fixed paraffin-embedded (FFPE) of NPP and PTT. The *n* = 32 paraffin-embedded samples (Additional File 1: Table S4) were obtained from the Pathology Unit at Hospital Joan XXIII in Tarragona. The extraction was performed using the MagMAX FFPE DNA/RNA Ultra Kit (Applied Biosystems) according to the manufacturer’s protocol.

### In vitro PCa cell experiment: transfection with miRNA inhibitors and gene silencer

The androgen-sensitive PCa cell line (22Rv1) and the histologically normal prostate epithelial cell line (RWPE-1) were purchased from Sigma-Aldrich (Barcelona, Spain). 22Rv1 cells were cultured in RPMI 1640 medium (Merck KGaA, Darmstadt, Germany). RWPE-1 cells were cultured in keratinocyte serum-free medium, containing 5 µg/mL bovine pituitary extract and 5 ng/mL recombinant human epidermal growth factor (Gibco, Fischer Scientific S.L., Madrid, Spain). Cell cultures were supplemented with 10% FBS, 1% penicillin/streptomycin, and 5 µg/mL Plasmocin^®^ (Invivogen, Zaragoza, Spain).

For transfection with miRNA inhibitors, 22Rv1 cells were seeded in 12-well or 6-well plates at 49.429 cells/cm^2^ for RNA or protein analysis, respectively. Twenty-four hours later, the medium was removed, and cells were transfected with 5, and 15 nM miRNA inhibitors (hsa-miR-20a-5p miRCURY LNA miRNA Power Inhibitor (i20a-5p), and hsa-miR-106b-5p miRCURY LNA miRNA Power Inhibitor (i106b-5p), using Lipofectamine 2000, P3000 reagent, and Optimem (Thermo Fisher, Madrid, Spain) according to the manufacturer’s protocol.

For gene silencing assays, 22Rv1 cells were seeded in 24-well plates at 47.897 cells/cm^2^ density. After 24 h, the medium was removed, and the cells were transfected with 10, 25, and 50 nM of the *RORA* small interfering RNA against all isoforms (siRORA: Silencer Select Pre-designed siRNA RORA; s12103, Ambion, Thermo Fisher), using Lipofectamine 2000 and Optimem. A negative control inhibitor (iNC: Negative control A miRCURY LNA miRNA Power Inhibitor Control; Qiagen, Madrid, Spain) and a non-target control small interfering RNA (siNC: Silencer^®^Select Negative Control siRNA; Ambion, Thermo Fisher) were used for comparative analyses.

Cells were collected after 24 h of transfection for RNA analysis or after 48 h for protein analysis.

### Gene and miRNAs expression analysis in cell extracts

Total RNA was isolated from PCa cells using RNeasy Mini Kit (Qiagen, Bionova, Barcelona, Spain).

For gene expression, cDNA was synthesized from total RNA using the High-Capacity cDNA reverse transcription kit (Applied Biosystems, Foster City, CA, USA). qRT-PCR was performed on a QuantStudio 7 Pro (Thermo Fisher Scientific, Massachusetts, USA) using TaqMan Universal PCR Master Mix Fast Advanced (Applied Biosystems, Fisher Scientific S.L., Madrid, Spain) and the following TaqMan assays: *RORA* (covers all 4 isoforms) (Hs00536545_m1) and *TNF-α* (Tumor Necrosis Factor; hs99999043_m1). The thermal cycle conditions were: 50ºC for 2 min (Uracil-N glycosylase activation), 95ºC for 2 min (Polymerase activation), and 40 cycles of 95ºC for 1 s (denaturation) and 60ºC for 20 s (annealing/extension). Raw data were extracted by DA2 software. In the paraffin samples, C_T_ values for *RORA* gene expression were normalized to the expression of 2 housekeeping genes: *UBA52* (Hs02835948_m1) (Ubiquitin A-52 Residue Ribosomal Protein Fusion Product 1) and *B2M* (Hs00187842_m1) (Beta-2-Microglobulin) [26, 27], while in PCa cell lines, C_T_ values for the *RORA* gene expression were normalized to *PPIA* (Hs99999904_m1) (Peptidylprolyl Isomerase A) as housekeeping.

For miRNA expression, the miRCURY LNA Universal RT microRNA PCR, Polyadenylation, and cDNA Synthesis Kit (Qiagen, Bionova, Barcelona, Spain) was used for reverse transcription. qRT-PCR was performed on a QuantStudio 7 Pro (Thermo Fisher Scientific, Massachusetts, USA) using ExiLENT SYBR Green Master Mix (Qiagen, Bionova, Barcelona, Spain), and the following TaqMan assays were used: hsa-miR-20a-5p, hsa-miR-106b-5p, hsa-miR-93-5p, and hsa-miR-17-5p. Hsa-miR-423-5, hsa-miR-103a-3p, hsa-miR-191-5p, and hsa-miR-16-5p expressions were used to normalize C_T_ miRNA values. The thermal cycle conditions were: 95ºC for 2 min (Polymerase activation), 40 cycles of 95ºC for 10 s (denaturation), and 60ºC for 1 min (annealing/extension).

All results were analysed using the comparative C_T_ method (2^−∆∆Ct^) and the data were expressed as an n-fold difference relative to a calibrator sample.

### Western blotting

The concentrated EVs and homogenised 22Rv1 cells were ultrasonicated 3 times during 1 min at a frequency of 50 kHz with a UP 200s Ultraschallprozessor sonic processor (Hielscher Ultrasonics GmbH, Germany). Total protein was quantified using the Pierce™ BCA Protein Assay Kit (Thermo Fisher, Rockford, IL, USA).

#### EVs surface molecules characterization

10 µg of protein isolated from PPAT-EVs, as well as, 10 µg of extract from human adipocyte cells, were resuspended in reducing sample buffer, boiled for 5 min at 95ºC, loaded on 4–15% SDS-PAGE gels, and immunoblotted with polyclonal rabbit antibodies against EXOAB-CD9A1, EXOAB-CD81A-1, EXOAB-CD63A-1, EXOABHsp70A-1, EXOAB-TSG101-1 (System Biology, Palo Alto, CA, USA) and with mouse monoclonal antibody for α-tubulin (Invitrogen, Thermo Fisher Scientific) at 1/1000 dilution. HRP-conjugated goat anti-mouse or anti-rabbit (both from Pierce, Thermo Fisher Scientific) were used as secondary antibodies at 1/500 dilution.

#### RORA protein analysis

25 µg of protein from 22Rv1 cells was electrophoresed on a 10% SDS-PAGE and transferred onto nitrocellulose membranes, blocked, and incubated with Anti-RORA mouse monoclonal antibody (sc-518,081; Santa Cruz, Spain) at 1/500 dilution and Anti-β-Actin mouse monoclonal antibody (clone AC-74; Sigma-Aldrich, Germany) at 1/1500 dilution. The purified Goat anti-Mouse IgG (H + L) HRP-Conjugate was used as a secondary antibody (Pierce/Thermo Fisher) at 1/2000 dilution.

In both cases, chemiluminescent western blot detection was developed with SuperSignal West Femto chemiluminescent substrate (Pierce Biotechnology, Boston, MA, USA) except for β-actin, which was developed with West Pico (Pierce Biotechnology). Images were quantified with VersaDoc Imaging System using Quantity One software (Bio-Rad, Barcelona, Spain) following the manufacturer’s instructions, and normalized to the amount of β-actin and α-tubulin for RORA protein analysis, and EVs characterization respectively.

### Cell proliferation assay

Cell proliferation was determined using the Cell Counting Kit-8 (CCK-8) (Sigma-Aldrich, Madrid, Spain). 22Rv1 cells were seeded at 26.316 cells/cm^2^ on 24-well plates and incubated for 24 h. Cells were then transfected with 15 nM miRNA inhibitors (i106b-5p or i-20a-5p) and/or 50 nM si*RORA*, as commented above. iNC and siNC were included as controls. Cell viability was measured at 24, 48, 72, and 96 h. At each time point, the culture medium was discarded, and 500 µl of fresh culture media was added, mixed with 50 µl of the CCK-8 reagent, and incubated at 37ºC for 2 hours. The media was then collected, and the absorbance was measured at a wavelength of 450 nm using a multi-mode microplate reader (BioTek).

### Statistical analysis

For the pilot miRNAS microarray study, sample size was calculated following the Mdanderson bioinformatic software (https://bioinformatics.mdanderson.org/MicroarraySampleSize/*).* Briefly, based on the measurement of 752 miRNAs, considering 50 false positives, 2-fold-change differences between sample groups, a standard deviation of 0.5, and an estimated power analysis of 0.9, the minimum sample size required was calculated to be 4 patients in each group.

For miRNA and gene validation analysis in paraffin samples, the sample size was calculated using G*Power 3.1.9.7. Assuming a change of 2-fold between groups and similar group variances, with an average power > 90% and a false discovery rate of 5%, a minimum of 19 patients was calculated to be needed in each group. The normality of the anthropometric and clinical variables was analysed with the Shapiro-Wilk test. The data is shown as the median with an interquartile range.

Clinical variables with non-normal distributions are reported as medians and interquartile ranges. To compare miRNA expression levels between PVAT (*n* = 17) and PPAT (*n* = 24) samples, only 16 samples were matched pairs from the same patients. Given the absence of a pre-and-post intervention effect, paired data analysis was deemed inappropriate. Therefore, the Mann-Whitney U test was employed to assess differences between the two patient groups.

In vitro experimental results are presented as the mean and standard error of the mean (SEM) of 3–4 independent experiments. Differences were tested with the unpaired two-tailed Student’s t-test. Statistical analyses were performed using the Statistical Package for the Social Sciences, version 22 (SPSS, Chicago, IL). GraphPad Prism 7.0 was used for the box plot representation.

## Results

### EVs from peritumoral adipose tissue are actively internalized by PCa cells

**Fig. 1:**
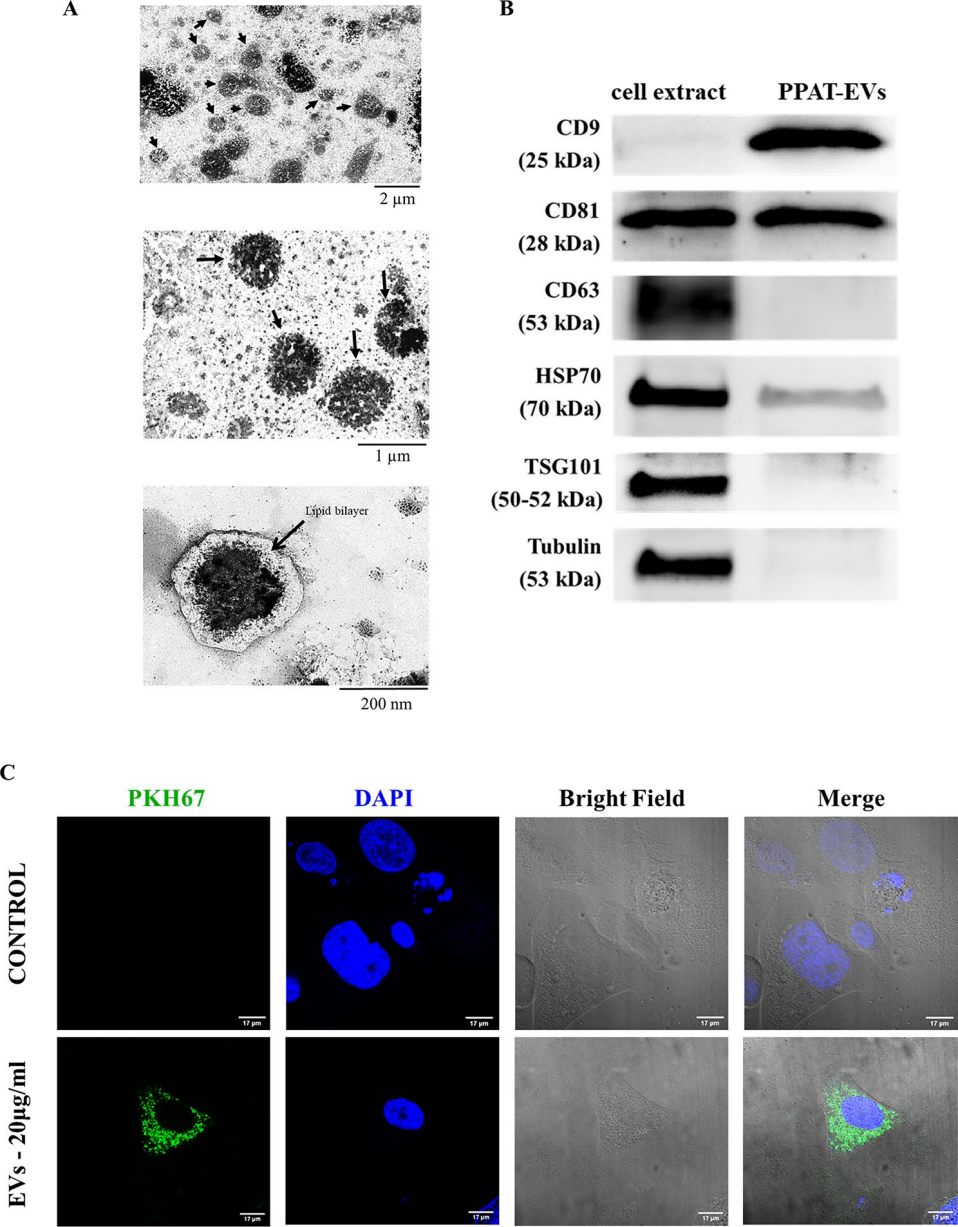
Characterization of isolated EVs. **A** Analysis of isolated extracellular vesicles (EVs) by transmission electron microscopy at different magnifications. **B** Western blot image of protein extracts prepared from isolated PPAT-derived EVs (PPAT-EV) and total adipocyte cell extract; tested with the following antibodies: CD9, CD81, CD63, HSP70, TSG101, and tubulin. (The uncropped Western Blot image is available in Additional File 2: Figure S1). **C** PPAT-EVs (20 µg/ml) uptake by PCa cells captured by confocal spectral microscope. PKH67 (green dye) labelled PPAT-EVs and DAPI (blue dye) labelled the cellular nucleus

To investigate whether EVs are secreted by PPAT and play a role in the communication with PCa cells, we purified small/medium sized EVs from the supernatant of PPAT explants after overnight culture (**Additional File 1: Table **S1 and S2). Isolated EVs were observed under TEM and showed the characteristics of small EVs, with a typical appearance and diameter ranging from 30 to 200 nm (Fig. 1A). Enrichment for EVs marker CD9, CD81, and the absence of the cell-specific marker tubulin was demonstrated by Western blot (Fig. 1B). The detailed results of immunoblotting are shown in Additional File 2: Figure S1.

To examine if 22Rv1 PCa cells might be targets of PPAT-EVs, a lipid-associating fluorescent dye, PKH67, was used to label EVs preparations and then incubated with PCa cells. EVs uptake was observed 1 h after treatment and was found to accumulate in PCa cells over time (Fig. 1C). Collectively, we showed that PPAT cells secrete EVs, which are actively incorporated *in vitro* by PCa cells.

### EVs derived from human PPAT revealed a unique miRNA profile

**Fig. 2:**
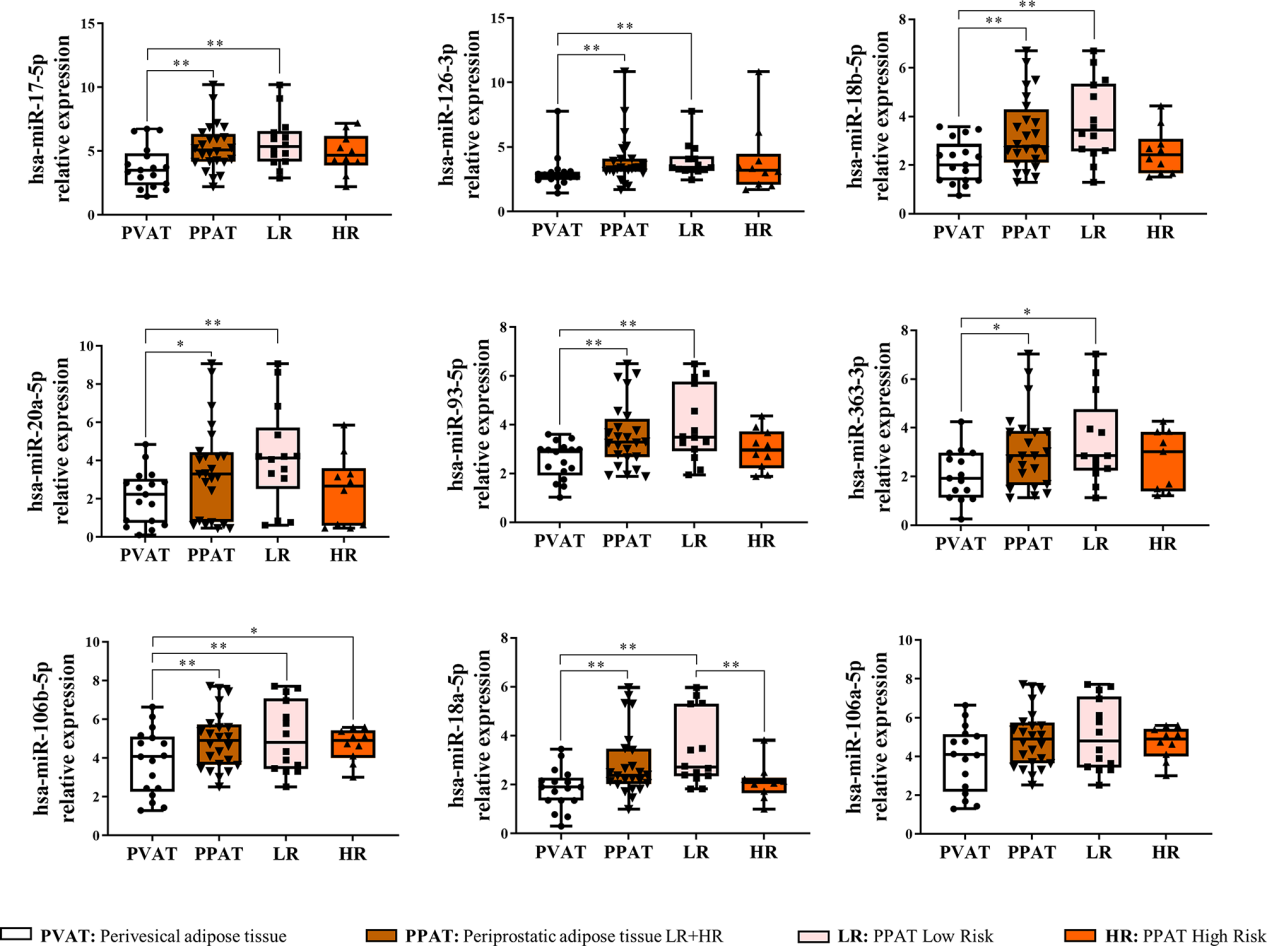
EVs-derived miRNAs are differentially expressed in PPAT from patients with prostate cancer. Box plots showing median, quartiles, and extreme values of relative expression of the nine discriminatory miRNAs in PPAT-EVs compared to PVAT-EVs. Adipose tissue samples were segregated according to ISUP grade in LR (Low-Risk; ISUP I and II) and HR (High-Risk; ISUP III, IV, and V). Symbols: *indicates significant differences, *p-value < 0.05; **p-value < 0.01

We searched the miRNA contained in EVs to find epigenetic regulators of PCa progression. The miRNA-profile search was divided into two phases: the initial pilot phase using AT from 4 PCa patients (Additional File 1: Table S1) and the validation phase using AT from 25 PCa patients (Additional File 1: Table S2). Thus, we first analysed miRNA expression from PPAT-EVs and PVAT-derived EVs (PVAT-EVs) samples using a qRT-PCR array of 752 miRNA target onco-miRNAs (Additional File 3: Raw data pilot study). Ten miRNAs were differentially expressed in PPAT-EVs vs. PVAT-EVs *(p-value* ≤ 0.05, C_T_ < 35 and expression values ≥ 1.8-fold or ≤ -1.8-fold): hsa-miR-18a-5p, hsa-miR-20a-5p, hsa-miR-363-3p, hsa-miR-18b-5p, hsa-miR-15a-5p, hsa-miR-93-5p, hsa-miR-17-5p, hsa-miR-15b-5p, hsa-miR-106a-5p, and hsa-miR-106b-5p. Moreover, the hsa-miR-126-3p with *p* < 0.1 was also selected because it met the requirements. Hence, these eleven selected miRNAs were further validated using a larger sample size (Additional File 1: Table S2). Analysis of the expression patterns of these eleven selected miRNAs revealed significant differences in 8 of them: hsa-miR-17-5p, hsa-miR-126-3p, hsa-miR-18b-5p, hsa-miR-20a-5p, hsa-miR-93-5p, hsa-miR-363-3p, hsa-miR-106b-5p, and hsa-miR-18a-5p when comparing PVAT-EVs vs. PPAT-EVs, and hsa-miR-106a-5p (*p* = 0.006) was close to significance (Fig. 2) (see C_T_ values in Additional File 3: Raw data validation study). When comparing the miRNA content of PVAT-EVs in terms of risk, the following miRNAs were up-regulated in low-risk PPAT-EVs: hsa-miR-17-5p, hsa-miR-126-3p, hsa-miR-18b-5p, hsa-miR-20a-5p, hsa-miR-93-5p, hsa-miR-363-3p, hsa-miR-106b-5p, and hsa-miR-18a-5p. While when comparing PVAT and high-risk PPAT-EVs, hsa miR-106b-5p was found significantly reduced. Hsa-miR-18a-5p was the only differentially expressed between PPAT-low risk vs. PPAT-high risk. No significant differences were detected for hsa-miR-106a-5p (Fig. 2).

Differences were observed when samples were segregated by ISUP grade. Between ISUP II and PVAT-EVs, significant differences were found for hsa-miR-17-5p, hsa-miR-126-3p, hsa-miR-18b-5p, hsa-miR-93-5p, and hsa-miR-18a-5p. Between ISUP I and PVAT-EVs, significant differences were observed for hsa-miR-18b-5p and hsa-miR-18a-5p. Additionally, between ISUP III and PVAT-EVs, significant differences were found for hsa-miR-106b-5p and hsa-miR-93-5p. No significant differences were found among the different ISUP grades (Additional File 4: Figure S2).

### RAR related orphan receptor A (*RORA*) gene is a common target of the putatively deregulated EVs-derived miRNAs

**Fig. 3:**
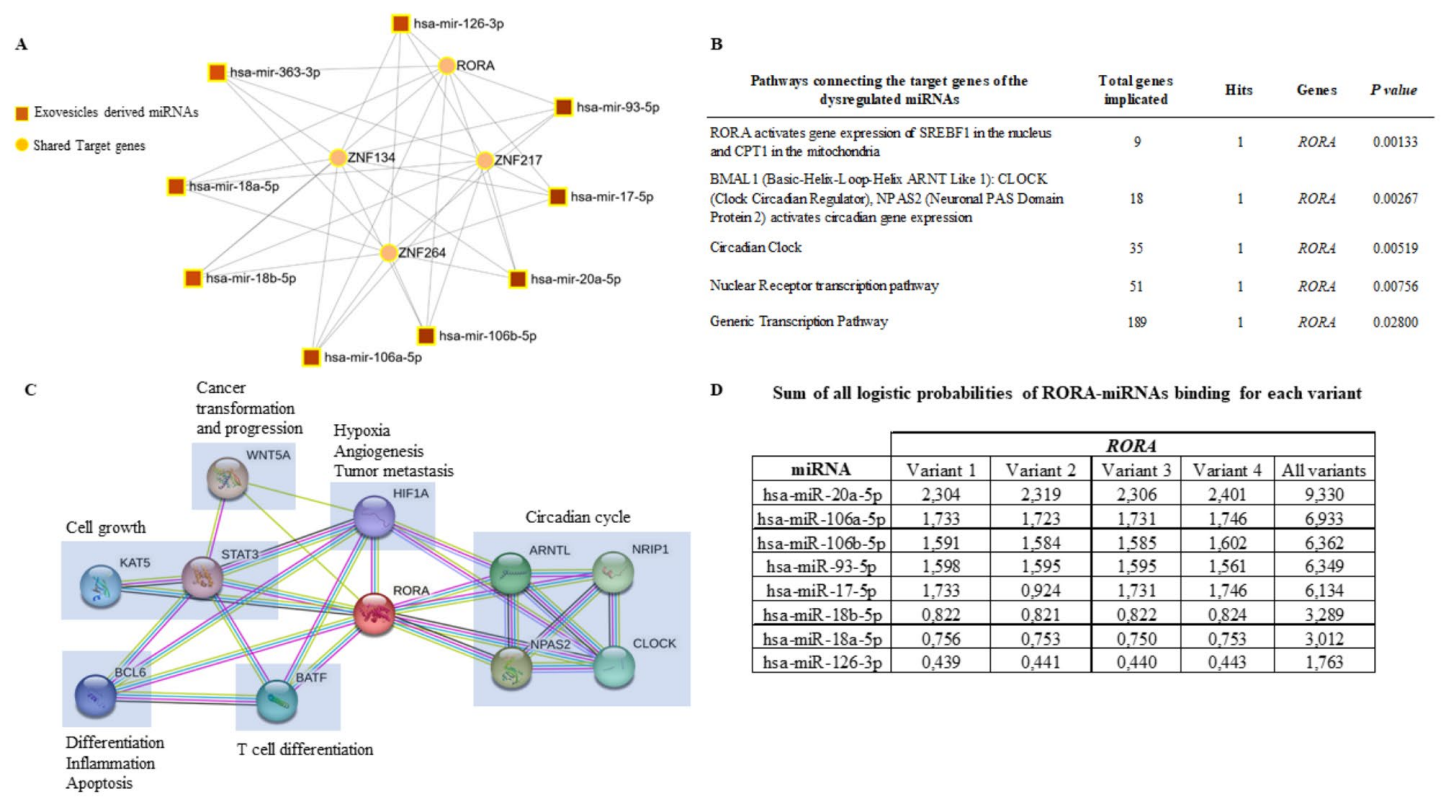
Gene-target and protein pathway regulatory networks of the selected miRNA. *In silico* analysis. **A** miRNet analysis showing that *RORA*,* ZNF134*,* ZNF217* and *ZNF264* genes were identified as common target of the putative miRNA-regulated pathways according to miRTarBase v8.0 and TarBase v8.0. **B** Reactome pathway analysis displaying the significant pathways connecting target genes of the dysregulated miRNAs. **Legend**. Total genes implicated: number of genes annotated in the Reactome; Hits: Number of genes resulting from the miRNet analysis involved in the corresponding pathway; Genes: Genes involved. **C** RORA protein-protein interacting network performed by STRING analysis. Network representing protein-protein associations (blue interactions are known from curated databases, pink is known experimentally determined, green is text mining and black are co-expression) (See Additional File 5: Figure S3 for more detailed information of each protein in the node). **D** Table illustrating the logistic probabilities of *RORA*-miRNA binding for each *RORA* gene variant (See Additional File 6: Figure S4 for more detailed information of each miRNA seed-gene binding site)

To better understand the function and mechanism of deregulated PPAT-EVs-derived miRNA in gene function, we searched for putative miRNA-target interactions using miRNet analysis software. The program was directed to identify target genes according to miRTarBase v8.0 and TarBase v8.0. After evaluating the 9 deregulated miRNAs, the software found that this combination was involved in the post-transcriptional regulation of 4 putative key genes: *RORA* and 3 Zinc Finger Proteins (*ZNF134*, *ZNF217* and *ZNF264*) (Fig. 3A). Based on this, we used Reactome software to examine the signalling pathways in which these genes might be engaged. Interestingly, we observed that the *RORA* gene was consistently identified in most of the most significant deregulated pathways (Fig. 3B), selecting this gene as a common target of the putatively deregulated PPAT-EVs-derived miRNAs. By analysing the STRING protein-protein interaction data base, we searched for its putative protein interaction network. The STRING search results showed 10 putative proteins with interaction scores between 0.9 and 1 that the model considered to be true. The 10 proteins were mainly related to cell growth (KAT5, STAT3), differentiation, inflammation, and apoptosis (BCL6), T cell differentiation (BATF), hypoxia, angiogenesis, and tumour metastasis (HIF1A), and circadian cycle (ARNTL, Basic Helix-Loop-Helix ARNT Like 1; NRIP1, Nuclear Receptor Interacting Protein 1; NPAS2, Neuronal PAS Domain Protein 2; CLOCK, Clock Circadian Regulator) (Fig. 3C and Additional File 5: Figure S3). We then checked the prediction of the deregulated miRNAs binding sites in the *RORA* gene using the STarMir web server. Binding sites for 8/9 miRNAs were detected in all 4 *RORA* gene variants, but no binding was uploaded for miR-363-3p. This is shown in Fig. 3D as the sum of all logistic probability of binding (LogitProb) of all 4 *RORA* gene variants in the 3’UTR seed region of each miRNA (see detail binding sites in Additional File 6: Figure S4). Thus, hsa-miR-20a-5p, hsa-miR-106b-5p, hsa-miR-93-5p, and hsa-miR-17-5p were selected as the most relevant regulatory miRNAs of the *RORA* gene according to the best LogitProb obtained and considering the different expression patterns of miRNA content in PPAT- EVs and PVAT- EVs.

### Expression of *RORA* gene and putatively regulating miRNAs in PCa human samples

The expression levels of the *RORA* gene and the expression levels of the most relevant miRNAs (hsa-miR-20a-5p, hsa-miR-106b-5p, hsa-miR-93-5p, and hsa-miR-17-5p) were uploaded from prostate tumour tissues (PTT) and non-pathogenic prostate tissues (NPP) obtained in the CancerMIRNome database (http://bioinfo.jialab-ucr.org/CancerMIRNome/).

**Fig. 4:**
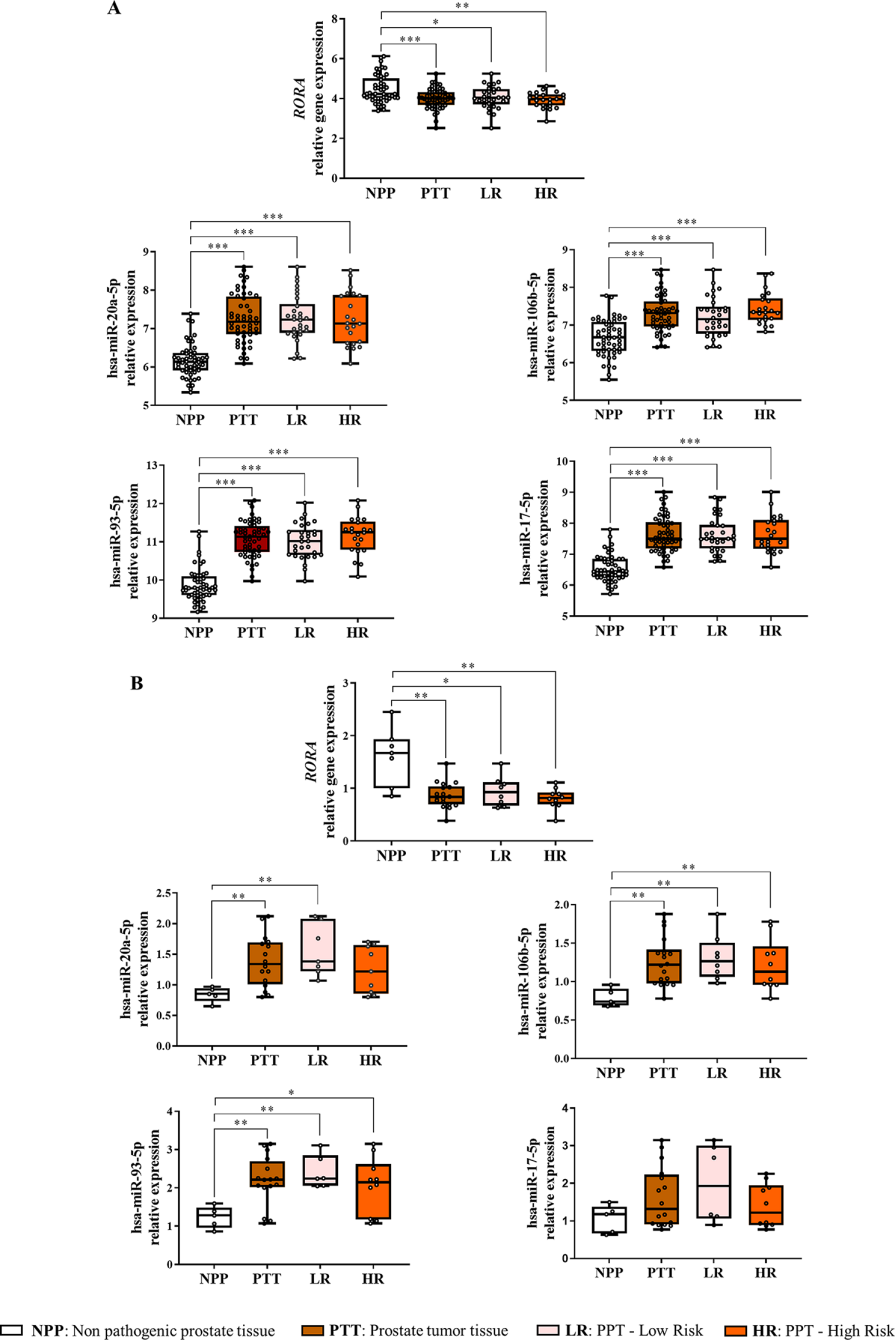
*RORA* gene and miRNAs expression in PCa tissues. **A** *RORA* and miRNAs expression level analysis in non-pathogenic prostate tissue (NPP) vs. PCa tissue samples (PTT) classified as LR (Low-Risk; ISUP I and II) and HR (High-Risk; ISUP III, IV, and V) using CancerMIRNome database (the y-axis relative units are: log_2_CPM = Counts per Million of gene reads/total reads x 10^6^). **B** *RORA* and miRNAs expression level analysis in NPP vs. PTT classified as LR (Low- Risk; ISUP I and II) and HR (High-Risk; ISUP III, IV and V) using human prostate tissue paraffin blocks. Each box shows the median, quartiles, and extreme values. Symbols: * indicates significant differences, *p-value < 0.05; **p-value < 0.01; ***p-value < 0.001

Anthropometric and clinical characteristics from the CancerMIRNome patient database are shown in Additional File 1: Table S3. Paired samples of PTT and NPP tissue from 52 selected patients were retrieved from the PRAD (Prostate Adenocarcinoma) project included in the TCGA. When analysing *RORA* gene expression in the selected samples, significantly lower expression levels were observed in PTT samples compared to NPP tissue. This reduction in the *RORA* gene expression was evident when comparing NPP to both PTT-low-risk and PTT-high-risk samples (Fig. 4A). However, no differences were observed in *RORA* gene expression levels between low and high-risk PTT (Fig. 4A). Interestingly, our analysis of the expression of selected miRNAs (hsa-miR-20a-5p, hsa-miR-106b-5p, hsa-miR-93-5p, and hsa-miR-17-5p) in 52 paired CancerMIRNome samples revealed that the expression patterns in NPP vs. PTT tissues closely mirrored those observed in PVAT-EVs vs. PPAT-EVs samples (Fig. 4A).

We then validated the in-silico results obtained from CancerMIRNome for the *RORA* gene expression and selected miRNAs in 32 paraffined tissue samples from PCa patients (Additional File 1: Table S4). The results confirmed the down-regulation of the *RORA* gene expression levels in PTT compared to paired NPP tissue. Regarding miRNA expression, the upregulation of hsa-miR-20a-5p, hsa-miR-106b-5p, and hsa-miR-93-5p was corroborated when comparing NPP to PTT, NPP to PTT-low-risk, and NPP to PTT-high-risk. However, no significant differences were detected between NPP and PTT-high-risk for hsa-miR-20a-5p. Additionally, no differences were observed for hsa-miR-17-5p across the sample comparisons (Fig. 4B).

### *RORA* is a target gene of hsa-miR-20a-5p and hsa-miR-106-5p

**Fig. 5:**
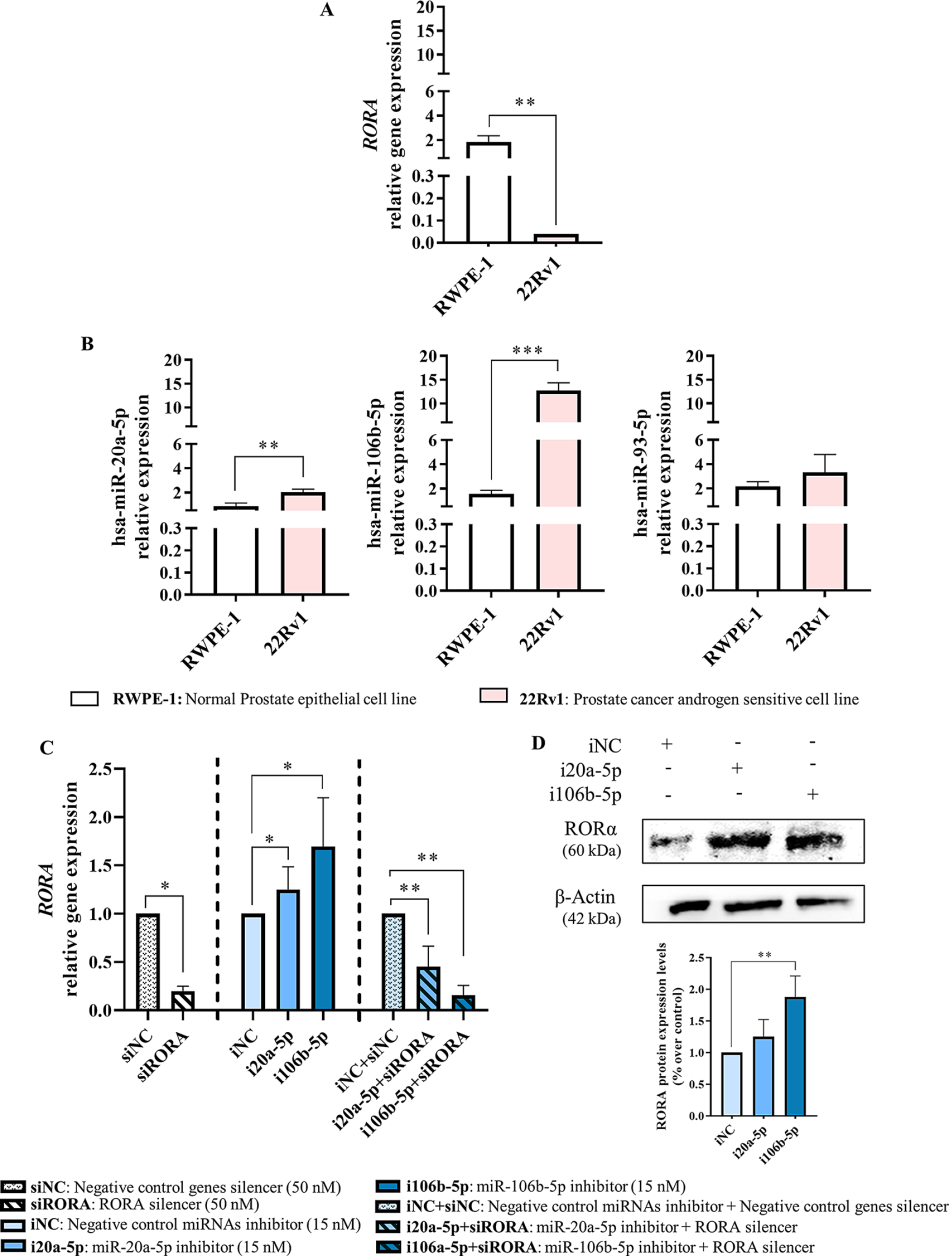
hsa-miR-20a-5p and hsa-miR-106b-5p regulate the *RORA* gene and protein expression in PCa cells. **A** Study of *RORA* and **B** miRNAs expression in histologically normal prostate epithelial cell line (RWPE-1) and the androgen-sensitive PCa cell line (22-Rv1). **C** *RORA* gene expression levels in 22Rv1 transfected with siRORA, miR-20a-5p inhibitor (i20a-5p) or miR-106b-5p inhibitor (i106b-5p) and co-transfected with siRORA and either i20a-5p or i106-5p. **D** RORA protein expression in 22Rv1 cells transfected with i20a-5p or i106b-5p inhibitor. A representative Western blot is presented (top). The membranes were tested with the corresponding antibodies. Expression levels were normalized to β-actin, then miRNAs expression levels were normalized to negative control inhibitor (iNC). Full-length blot and gel are presented in Additional File 8: Figure S6. Working concentration for siRORA was 50 nM, and for i20a-5p or i106b-5p was 15nM. Symbols: * indicates significant differences, *p-value < 0.05; **p-value < 0.01; ***p-value < 0.001

To investigate whether hsa-miR-20a-5p, hsa-miR-106b-5p, hsa-miR-93-5p, and hsa-miR-17-5p target the *RORA* gene, we utilized the RWPE-1 cell line as a control, representing healthy prostate epithelial cells. Additionally, we used the 22Rv1 cell line, an androgen-sensitive PCa cell model, which closely mimics the i*n vivo* conditions of our samples. This selection is pivotal, as all our PPAT samples are derived from androgen-sensitive tumours, thereby ensuring an accurate replication of the relevant biological context. First, we checked *RORA* gene expression in both cell lines, and we found that it was significantly down-regulated in the 22Rv1 PCa cell line compared to the control RWPE-1 cell line (Fig. 5A). Then, we analysed the expression levels of hsa-miR 20a-5p, hsa-miR-106b-5p, hsa-miR-93-5p, and hsa-miR-17-5p in both cell lines. Expressions of hsa-miR 20a-5p and hsa-miR-106b-5p were found to be significantly upregulated in the 22Rv1 cell line compared to RWPE-1 cell line, whereas no significant differences were observed for hsa-miR-93-5p (Fig. 5B). The expression of hsa-miR-17-5p showed very high Ct values (> 37) and was considered not expressed.

We then selected the 22Rv1 cell line and the significantly up-regulated miRNAs (hsa-miR-20a-5p and hsa-miR-106b-5p) (Fig. 5B) to validate our in-silico findings that suggest a link between these miRNAs and *RORA* gene expression regulation. To evaluate this hypothesis, using an effective dose of gene silencer (50 nM), we found that *RORA* gene expression was significantly decreased (Additional File 7: Figure S5A). Then, we transiently inhibited hsa-miR 20a-5p (i20a-5p) and hsa-miR-106b-5p (i106b-5p) using an effective miRNA inhibitor dose (15 nM) (Additional File 7: Figure S5B) and we detected a significant upregulation of *RORA* mRNA expression (Fig. 5C). Moreover, to further evaluate the role of these miRNAs in *RORA* mRNA expression, we inhibited *RORA* gene (siRORA) and hsa-miR-20a-5p/hsa-miR106b-5p simultaneously; and we found that when *RORA* gene and hsa-miR-20a-5p were simultaneously inhibited (i20a-5p + siRORA), *RORA* gene expression significantly decreased compared with cells treated with iNC + siNC pointing out that hsa-miR-20a-5p may have a modulatory role over *RORA* gene expression. Simultaneous inhibition of the *RORA* gene and hsa-miR-106-5p (i106b-5p + siRORA) also show a significant effect as compared to control iNC + siNC. Similar data was obtained on RORA protein expression when i106b-5p was used, but only a trend (*p* = 0.17) was observed for i20a-5p (Fig. 5D) (western blot details can be found in Additional File 8: Figure S6). No protein expression was detected when the *RORA* gene was silenced or when both *RORA* gene and hsa-miR-20a-5p/hsa-miR106b-5p were co-inhibited simultaneously.

### *RORA* gene inhibits inflammation and proliferation in PCa cells through hsa-miR-20a-5p

**Fig. 6:**
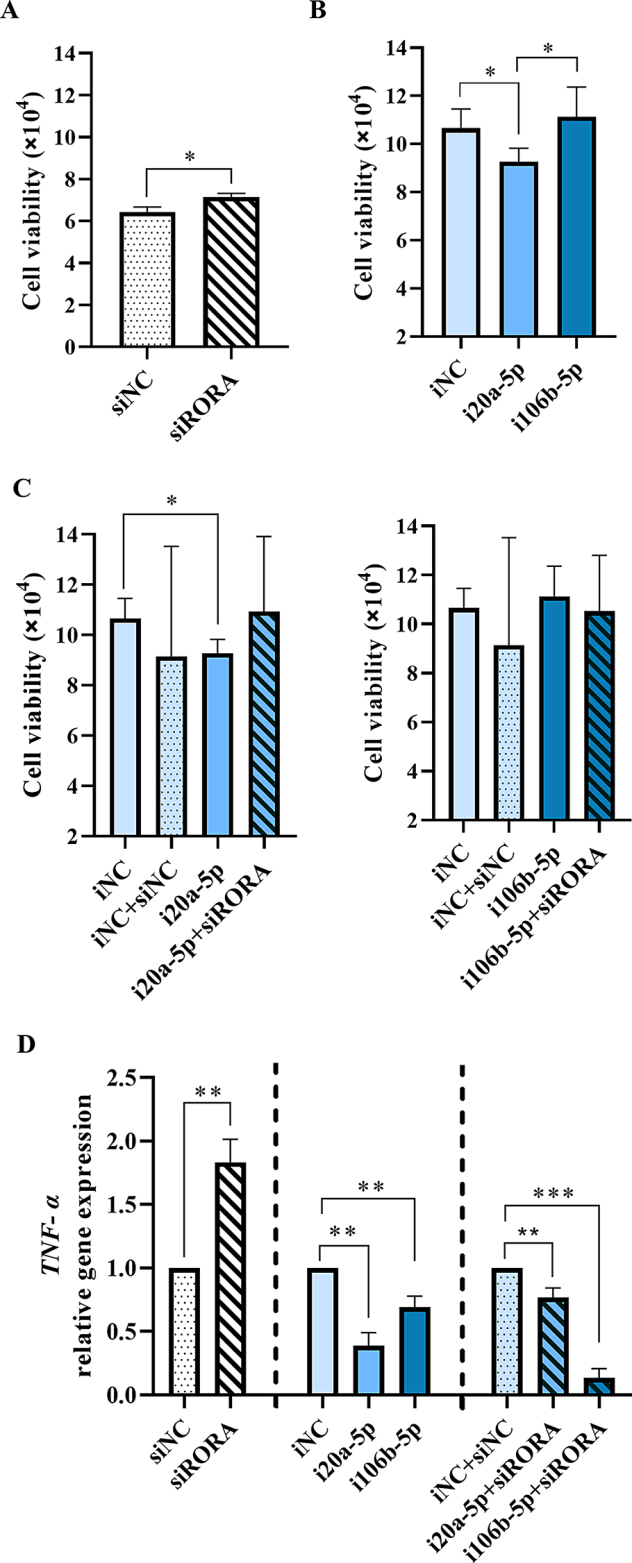
hsa-miR-20a-5p alters proliferation and inflammation by targeting *RORA* gene in 22Rv1 PCa cells. **A-C** Cell proliferation studies in 22Rv1 after transfection with siRORA at 96 h, and with miR-20a-5p inhibitor (i20a-5p) or miR-106b-5p inhibitor (i106b-5p) at 72 h or in combination (siRORA plus corresponding miRNA inhibitor). **D** *TNF-α* mRNA expression levels in 22Rv1 transfected with siRORA, miR-20a-5p inhibitor (i20a-5p) or miR-106b-5p inhibitor (i106b-5p) alone or in combination. Symbols: * indicates significant differences, *p-*value* < 0,05; **p-value < 0.01; ***p-value < 0.001

To investigate whether PPAT-EVs derived miRNAs, hsa-miR-106b-5p and hsa-miR-20a-5p, could regulate inflammation and proliferation in the 22Rv1 PCa cell line by targeting *RORA* gene, we silenced simultaneously and individually *RORA* gene expression and hsa-miR-106b-5p/hsa-miR-20a-5p correspondingly (Fig. 6).

The effect of hsa-miR-20a-5p and hsa-miR-106-5p on PCa cell proliferation through *RORA* gene was evaluated (Additional File 9: Figure S7). First, we observed that silencing the *RORA* gene with siRORA, induced a significantly increased 22Rv1 cell proliferation (Fig. 6A). Then, we inhibited hsa-miR-20a-5p, and a decrease in cell proliferation was observed, while hsa-miR-106b-5p inhibition did not affect cell proliferation (Fig. 6B). When we simultaneously inhibited the *RORA* gene and hsa-miR-20a-5p (siRORA + i20a-5p), cell proliferation was partially increased, indicating that hsa-miR-20a-5p regulates PCa cell proliferation through the *RORA* gene (Fig. 6C). Inhibition of the *RORA* gene and hsa-miR-106b-5p (i106b-5p + siRORA) did not show any effect on 22Rv1cell proliferation (Fig. 6C).

*RORA* gene silencing resulted in increased *TNF-α* mRNA gene expression, which was decreased when hsa-miR-106b-5p and hsa-miR-20a-5p were inhibited (Fig. 6D). The hsa-miR-20a-5p inhibition (i20a-5p) had a higher reduced expression effect on *TNF-α* mRNA levels, although no significant differences were observed when comparing the inhibitory effect on *TNF-α* between hsa-miR-106b-5p and hsa-miR-20a-5p (Fig. 6D). Interestingly, when the *RORA* gene expression and hsa-miR-20a-5p were inhibited simultaneously (i20a-5p + siRORA), *TNF-α* mRNA expression was partially rescued (Fig. 6D), while no compensatory effect was observed when hsa-miR-106b-5p and the *RORA* gene were silenced simultaneously (i106b-5p + siRORA) (Fig. 6D).

## Discussion

Trafficking of EVs in the TME has been found to be altered during cancer progression [28]. In this microenvironment, PPAT-EVs have been demonstrated to modulate cancer features [19, 20], partly driven by their miRNA contents; an issue scarcely investigated in the context of PCa AT microenvironment. Therefore, in this study, we evaluated, for the first time to our knowledge, the involvement of miRNAs contained in EVs secreted by human PPAT in the progression and aggressiveness of PCa tumour.

We used a battery of tests to ensure that the isolated miRNAs originated from PPAT- EVs, including protein expression of EVs surface markers, as recommended by the ISEV [29], and size characterization, which revealed uniformity in size distribution.

Microarray analysis identified 9 differentially expressed miRNAs derived from PPAT-EVs compared to those miRNAs derived from PVAT-EVs samples (hsa-miR-17-5p, -126-3p, -18b-5p, -20a-5p, -93-5p, -363-3p, -106b-5p, -18a-5p, and − 106a-5p). Remarkably, all miRNAs were significantly more abundant in the PPAT-EVs samples than in the PVAT-EVs samples. Interestingly, when PPAT-EVs were analysed based on PCa aggressiveness, no differences were observed in miRNA content between low-risk and high-risk PPAT-EVs. However, miRNA expression in low-risk PPAT-EVs was significantly higher compared to PVAT-EVs, a difference that was not observed when comparing high-risk PPAT-EVs to PVAT-EVs. The higher expression of these miRNAs in low-risk PPAT-EVs, especially in lower ISUP grades, may indicate an active suppression of tumour progression by these miRNAs. As the tumour becomes more aggressive (higher ISUP grades), it might down-regulate these miRNAs to evade their suppressive effects. Overall, this observation reinforces the impact of the tumour on its microenvironment, and particularly the effect of peritumoral AT, as suggested in other types of tumours like breast [30].

The miRNA network analysis of selected PPAT-EVs miRNAs identified four potential shared target genes: RAR Related Orphan Receptor A (RORA) and three Zinc Finger Proteins (ZNF134, ZNF217, and ZNF264). In addition, pathway enrichment analysis identified the *RORA* gene as a common denominator of all the putatively deregulated pathways. The *RORA* gene or also called NR1F1 (nuclear receptor subfamily 1, group F, member 1) is a transcription factor that plays a critical role in the regulation of various biological processes, including circadian rhythm, metabolism, and inflammation [31]. Recent studies have suggested that RORA may also be involved in PCa progression [32], even a RORA polymorphism rs17191414 has been associated with PCa risk, however, this data needs to be validated in other cohorts [33].

*RORA* gene is composed of 15 exons, located in the middle of chromosome 15q22.2. As endogenous ligands, cholesterol and derivatives have been appointed [31, 34]. This gene is generated by splicing, giving rise to four isoforms (α1 to α4) in humans that differ in the N-terminal region [35–37]. Variant 1 (α1) is expressed in healthy breast, brain, prostate, liver, and ovarian tissue. In the prostate, the main variants found are α1 and α4 [38].

Our results demonstrated that the most relevant PPAT-EV deregulated miRNAs (hsa-miR20a-5p, -106b-5p, -93-5p, and − 17-5p), selected according to the best logistic seed binding probability and by their expression patterns in PPAT- EVs, can all bind to the 4 RORA variants in the 3’UTR region. Furthermore, their expression levels were upregulated in PCa tissue samples compared to non-pathogenic prostate tissue samples, as retrieved from the CancerMIRNome database, and corroborated by our cohort of paraffin embedded PCa samples. The expression patterns of hsa-miR-20a-5p and hsa-miR-106b-5p were also replicated in the 22Rv1 cancer cell line, showing higher expression than in the healthy RWPE-1 cell line. Additionally, *RORA* gene expression was reduced in prostate tumour tissues, and in PCa cells compared to normal tissue or healthy prostate cells, a feature confirmed by other authors [38].

Given these findings, we conducted further in vitro experiments to explore the miRNA regulatory effect on the *RORA* gene in the 22Rv1 PCa cell line. This cell line was chosen to accurately replicate the effects of the PPAT-EVs within a relevant biological context, as all our PPAT samples originate from androgen-sensitive tumours, and 22Rv1 cells derive from a primary prostate tumour and possess the androgen receptor. Using inhibitors of two deregulated miRNAs (hsa-miR-20a-5p and hsa-miR-106b-5p), we observed an increase in mRNA and protein levels of RORA in the androgen-sensitive 22Rv1 PCa cell line. Additionally, we observed a reduction in cell viability of the 22Rv1 cell line when using the hsa-miR-20a-5p inhibitor (i20a-5p). This reduction was partially rescued when the *RORA* gene was silenced, indicating that *RORA* gene expression is epigenetically regulated by hsa-miR-20a-5p, thus reducing the proliferative capacity of tumour cells. The involvement of the *RORA* gene in proliferation was further evidenced by the up-regulation of hsa-miR-1290 in PCa cell lines [39].

*RORA* gene expression has been associated with decreased proliferation and invasion because it inhibits the Wnt/β-catenin pathway, preventing the transformation and progression of some types of cancer, such as breast cancer [31] and negatively regulates genes such as c-jun, c-myc and cyclin D1 [36]. RORA’s pathway action can be through direct binding to DNA: canonical pathway; or by coupling to other molecules involved in the Wnt or p53 pathway: a non-canonical pathway [31, 40]. RORα1 variant has been demonstrated to have anti-proliferative activity, also affecting cell cycle progression in the DU145 androgen-dependent PCa cell line (by modulating p21 and cyclin A) as well as inhibiting the conversion of fatty acids into 5-5-Hydroxyeicosatetraenoic acid (responsible for the proliferative effect) [41].

RORA has been described as a negative regulator of the inflammatory response in several processes [42–44]. Thus, RORA expression has also been associated with anti-inflammatory capacity in primary human aortic cells [44] a finding consistent with the fact that its deletion confers pro-inflammatory characteristics by polarizing macrophages to M1 type [31, 42]. Moreover, RORA has been shown to decrease inflammation in breast cancer cells by inhibiting reactive oxygen species-mediated cytokine expression [45]. However, the better-known pathway by which RORA negatively regulates the inflammatory processes is via NF-ĸB (Nuclear Factor Kappa B Subunit 1) signalling pathway, involving the inhibition of TNF-α (Tumor Necrosis Factor alpha) [46, 47]. In the PCa context, we found that hsa-miR-20a-5p and hsa-miR-106b-5p may reduce inflammation by targeting *RORA* gene through regulation of *TNF-α* expression. In fact, we demonstrated that *RORA* gene silencing increases *TNF-α* gene expression, pointing out that RORA is regulating *TNF-α* gene expression. Interestingly, when hsa-miR-20a-5p and hsa-miR-106b-5p were inhibited, *TNF-α* gene expression was decreased, indicating that both miRNAs were involved in *TNF-α* gene regulation. However, only simultaneous co-inhibition of the hsa-miR-20a-5p and siRORA gene rescued *TNF-α* gene expression. Thus, this finding is in concordance with those observed in proliferation, suggesting for the first time to our knowledge that hsa-miR-20a-5p is involved in regulating proliferation and inflammation through targeting *RORA* gene in the 22Rv1 PCa cell line.

## Conclusions

We have identified deregulated miRNAs contained in EVs secreted by PPAT that target *RORA* gene, which has a role in the proliferation and inflammation of PCa cells, reinforcing the implications of PPAT in PCa aggressiveness, and revealing its potential for the development of new therapeutic strategies.

### Electronic supplementary material

Below is the link to the electronic supplementary material.

Supplementary Material 1

Supplementary Material 2

Supplementary Material 3

Supplementary Material 4

Supplementary Material 5

Supplementary Material 6

Supplementary Material 7

Supplementary Material 8

Supplementary Material 9

## Data Availability

All data generated or analysed during this study are included in this article, and its additional information files. The datasets underlying this article will be shared on reasonable request to the corresponding author.

## Abbreviations

ARNTL: Basic Helix-Loop-Helix ARNT Like 1
AT: Adipose Tissue
BATF: Basic Leucine Zipper ATF-Like Transcription Factor
BCL6: BCL6 Transcription Repressor
BCL-X1: B-cell lymphoma extra-large
CCL7: C-C Motif Chemokine Ligand 7
CCR3: CCL7 Chemokine Receptor
CCK-8: Cell Counting Kit
CEIm: Ethical Committee for Clinical Research
CLOCK: Clock Circadian Regulator
CT: Cycle Threshold
DA2: Design and Analysis Software v.2.6.0
DCTX: Docetaxel
FBS: Foetal Bovine Serum
FFPE: Formalin-Fixed Paraffin-Embedded
EVs: Exovesicles
HIF1A: Hypoxia Inducible Factor 1 Subunit Alpha
IL-6: Interleukin 6
iNC: Negative control A miRCURY LNA miRNA Power Inhibitor Control
ISEV: International Society for Extracellular Vesicles
ISUP: International Society of Urological Pathology
i20a-5p: hsa-miR-20a-5p miRCURY LNA miRNA Power Inhibitor
i106b-5p: hsa-miR-106b-5p miRCURY LNA miRNA Power Inhibitor
LogitProb: Logistic probability of binding
NF-kB: Nuclear Factor Kappa B Subunit 1
MMP-9: Matrix Metallopeptidase 9
mRNA: messenger RNA
NPAS2: Neuronal PAS Domain Protein 2
NR1F1: nuclear receptor subfamily 1, group F, member 1
NPP: Non-Pathogenic Prostate
NRIP1: Nuclear Receptor Interacting Protein 1
KAT5: Lysine Acetyltransferase 5
PCa: Prostate Cancer
qRT-PCR: Quantitative Real Time Polymerase chain reaction
PPAT: Periprostatic adipose tissue
PPAT-EVs: PPAT-derived exovesicles
PPIA: Peptidylprolyl Isomerase A
PRAD: Prostate Adenocarcinoma
PTT: Prostate Tumour Tissue
PVAT: Perivesical adipose tissue
PVAT-EVs: PVAT-derived exovesicles
RORA: RAR Related Orphan Receptor A gene
siNC: Silencer^®^Select Negative Control siRNA
SEM: Standard error of the mean
siRORA: Silencer Select Pre-designed siRNA RORA
STAT3: Signal Transducer And Activator Of Transcription 3
TCGA: The Cancer Genome Atlas
TGFα: Transforming Growth Factor Alpha
TEM: Transmission Electron Microscopy
TME: Tumour Microenvironment
TNF-α: Tumor Necrosis Factor alpha
TUBB2B: β-tubulin isoform 2B
TWIST1: Twist Family BHLH Transcription Factor 1
UTR: Untranslated region
ZNF134: Zinc Finger Protein 134
ZNF217: Zinc Finger Protein 217
ZNF264: Zinc Finger Protein 264

## References

[1] Bray F, Laversanne M, Sung H, Ferlay J, Siegel RL, Soerjomataram I, Jemal A. Global cancer statistics 2022: GLOBOCAN estimates of incidence and mortality worldwide for 36 cancers in 185 countries. CA Cancer J Clin. 2024;74:229–63. 10.3322/caac.21834 38572751

[2] Wang L, Lu B, He M, Wang Y, Wang Z, Du L. Prostate Cancer incidence and mortality: global status and temporal trends in 89 countries from 2000 to 2019. Front Public Health. 2022;10:811044. 10.3389/fpubh.2022.811044 PMC8888523 35252092

[3] Rebello RJ, Oing C, Knudsen KE, Loeb S, Johnson DC, Reiter RE, et al. Prostate cancer. Nat Rev Dis Primers. 2021;7:9. 10.1038/s41572-020-00243-0 33542230

[4] Mansinho A, Macedo D, Fernandes I, Costa L. Castration-resistant prostate cancer: mechanisms, targets, and treatment. Adv Exp Med Biol. 2018;1096:117–33. 10.1007/978-3-319-99286-0_7 30324351

[5] Anderson NM, Simon MC. The tumor microenvironment. Curr Biol. 2020;30:R921–25. 10.1016/j.cub.2020.06.081 PMC8194051 32810447

[6] Lo Iacono M, Modica C, Porcelli G, Brancato OR, Muratore G, Bianca P, et al. Targeting of the peritumoral adipose tissue microenvironment as an innovative antitumor therapeutic strategy. Biomolecules. 2022;12:702. 10.3390/biom12050702 PMC9138344 35625629

[7] Woo S, Cho JY, Kim SY, Kim SH. Periprostatic fat thickness on MRI: correlation with Gleason score in prostate cancer. AJR Am J Roentgenol. 2015;204:W43–7. 10.2214/AJR.14.12689 25539273

[8] Bhindi B, Trottier G, Elharram M, Fernandes KA, Lockwood G, Toi A (2012). Measurement of peri-prostatic fat thickness using transrectal ultrasonography (TRUS): a new risk factor for prostate cancer. BJU Int.

[9] Ribeiro R, Monteiro C, Cunha V, Oliveira MJ, Freitas M, Fraga A. Human periprostatic adipose tissue promotes prostate cancer aggressiveness in vitro. J Exp Clin Cancer Res. 2012;31:32. 10.1186/1756-9966-31-32 PMC3379940 22469146

[10] Laurent V, Gue A, Nieto L, Zaidi F, Majed B, Garandeau D (2016). Periprostatic adipocytes act as a driving force for prostate cancer progression in obesity. Nat Commun.

[11] Zhang Q, Sun L, jiang, Yang Z gang, Zhang G. ming, Huo R cha. Influence of adipocytokines in periprostatic adipose tissue on prostate cancer aggressiveness. Cytokine. 2016;85:148–56. 10.1016/j.cyto.2016.06.019 27371773

[12] la Civita E, Liotti A, Cennamo M, Crocetto F, Ferro M, Liguoro P, et al. Peri-prostatic adipocyte-released tgfβ enhances prostate cancer cell motility by upregulation of connective tissue growth factor. Biomedicines. 2021;9:1692. 10.3390/biomedicines9111692 PMC8615771 34829922

[13] Estève D, Roumiguié M, Manceau C, Milhas D, Muller C. Periprostatic adipose tissue: a heavy player in prostate cancer progression. Curr Opin Endocr Metab Res. 2020;10:29–35.

[14] Liotti A, La Civita E, Cennamo M, Crocetto F, Ferro M, Guadagno E, et al. Periprostatic adipose tissue promotes prostate cancer resistance to docetaxel by paracrine IGF-1 upregulation of TUBB2B beta-tubulin isoform. Prostate. 2021;81:407–17. 10.1002/pros.24117 PMC8251776 33734457

[15] Altuna-Coy A, Ruiz-Plazas X, Sánchez-Martin S, Ascaso-Til H, Prados-Saavedra M, Alves-Santiago M, et al. The lipidomic profile of the tumoral periprostatic adipose tissue reveals alterations in tumor cell’s metabolic crosstalk. BMC Med. 2022;20:255. 10.1186/s12916-022-02457-3 PMC9386931 35978404

[16] Yáñez-Mó M, Siljander PRM, Andreu Z, Zavec AB, Borràs FE, Buzas EI (2015). Biological properties of extracellular vesicles and their physiological functions. J Extracell Vesicles.

[17] Welsh JA, Goberdhan DCI, O’Driscoll L, Buzas EI, Blenkiron C, Bussolati B, et al. Minimal information for studies of extracellular vesicles (MISEV2023): from basic to advanced approaches. J Extracell Vesicles. 2024;13:e12404. 10.1002/jev2.12404 PMC10850029 38326288

[18] Vanacore D, Boccellino M, Rossetti S, Cavaliere C, D’aniello C, Di Franco R, et al. Micrornas in prostate cancer: an overview. Oncotarget. 2017;8:50240–51. 10.18632/oncotarget.16933 PMC5564846 28445135

[19] Lazar I, Clement E, Dauvillier S, Milhas D, Ducoux-Petit M, LeGonidec S (2016). Adipocyte exosomes promote Melanoma aggressiveness through fatty acid oxidation: a novel mechanism linking obesity and cancer. Cancer Res.

[20] Jeurissen S, Vergauwen G, Van Deun J, Lapeire L, Depoorter V, Miinalainen I (2017). The isolation of morphologically intact and biologically active extracellular vesicles from the secretome of cancer-associated adipose tissue. Cell Adh Migr.

[21] Gernapudi R, Yao Y, Zhang Y, Wolfson B, Roy S, Duru N (2015). Targeting exosomes from preadipocytes inhibits preadipocyte to cancer stem cell signaling in early-stage breast cancer. Breast Cancer Res Treat.

[22] Reza AMMT, Choi YJ, Yasuda H, Kim JH. Human adipose mesenchymal stem cell-derived exosomal-miRNAs are critical factors for inducing anti-proliferation signalling to A2780 and SKOV-3 ovarian cancer cells. Sci Rep. 2016;6:38498. 10.1038/srep38498 PMC5143979 27929108

[23] Epstein JI, Egevad L, Amin MB, Delahunt B, Srigley JR, Humphrey PA (2016). The 2014 International Society of Urological Pathology (ISUP) consensus conference on Gleason grading of prostatic carcinoma: definition of grading patterns and proposal for a new grading system. Am J Surg Pathol.

[24] Vandesompele J, De Preter K, Pattyn ilip, Poppe B, Van Roy N, De Paepe A, et al. Accurate normalization of real-time quantitative RT-PCR data by geometric averaging of multiple internal control genes. Genome biology. 2002;3. 10.1186/gb-2002-3-7-research0034 PMC126239 12184808

[25] Rennie W, Liu C, Carmack CS, Wolenc A, Kanoria S, Lu J (2014). STarMir: a web server for prediction of microRNA binding sites. Nucleic Acids Res.

[26] Agell L, Hernández S, Nonell L, Lorenzo M, Puigdecanet E, De Muga S (2012). A 12-gene expression signature is associated with aggressive histological in prostate cancer: SEC14L1 and TCEB1 genes are potential markers of progression. Am J Pathol.

[27] Cuzick J, Swanson GP, Fisher G, Brothman AR, Berney DM, Reid JE (2011). Prognostic value of an RNA expression signature derived from cell cycle proliferation genes in patients with prostate cancer: a retrospective study. Lancet Oncol.

[28] Kalluri R. The biology and function of exosomes in cancer. J Clin Invest. 2016;126:1208–15. 10.1172/JCI81135 PMC4811149 27035812

[29] Lötvall J, Hill AF, Hochberg F, Buzás EI, Di Vizio D, Gardiner C, et al. Minimal experimental requirements for definition of extracellular vesicles and their functions: a position statement from the International Society for Extracellular Vesicles. J Extracell Vesicles. 2014;3:26913. 10.3402/jev.v3.26913 PMC4275645 25536934

[30] Liu S, Benito-Martin A, Pelissier Vatter FA, Hanif SZ, Liu C, Bhardwaj P, et al. Breast adipose tissue‐derived extracellular vesicles from obese women alter tumor cell metabolism. EMBO Rep. 2023;24:e57339. 10.15252/embr.202357339 PMC10702795 37929643

[31] Lee JM, Kim H, Baek SH. Unraveling the physiological roles of retinoic acid receptor-related orphan receptor α. Exp Mol Med. 2021;53:1278–86. 10.1038/s12276-021-00679-8 PMC8492739 34588606

[32] Sakellakis M (2022). Orphan receptors in prostate cancer. Prostate.

[33] Benna C, Helfrich-Förster C, Rajendran S, Monticelli H, Pilati P, Nitti D, et al. Genetic variation of clock genes and cancer risk: a field synopsis and meta-analysis. Oncotarget. 2017;8:23978–95. 10.18632/oncotarget.15074 PMC5410358 28177907

[34] Kallen JA, Schlaeppi J-M, Bitsch F, Geisse S, Geiser M, Delhon I (2002). X-Ray structure of the hRORα LBD at 1.63 Å: structural and functional data that cholesterol or a cholesterol derivative is the natural ligand of RORα. Struct (London).

[35] Roshan-Moniri M, Hsing M, Butler MS, Cherkasov A, Rennie PS (2014). Orphan nuclear receptors as drug targets for the treatment of prostate and breast cancers. Cancer Treat Rev.

[36] Park SC, Park IG, Kim H, Lee JM. N-terminal domain mediated regulation of ROrα1 inhibits invasive growth in prostate cancer. Int J Mol Sci. 2019;20:1684. 10.3390/ijms20071684 PMC6479703 30987323

[37] Matsuoka H, Michihara A. Identification of the rorα transcriptional network contributes to the search for therapeutic targets in atherosclerosis. Biol Pharm Bull. 2021;44:1607–16. 10.1248/bpb.b21-00426 34719639

[38] Zhu Y, McAvoy S, Kuhn R, Smith DI (2006). RORA, a large common fragile site gene, is involved in cellular stress response. Oncogene.

[39] Li Y, He J, Yu L, Yang Q, Du J, Chen Y, et al. Hsa-miR-1290 is associated with stemness and invasiveness in prostate cancer cell lines by targeting RORA. Andrologia. 2022;54:e14396. 10.1111/and.14396 35220610

[40] Du J, Xu R (2012). RORα, a potential tumor suppressor and therapeutic target of breast cancer. Int J Mol Sci.

[41] Moretti RM, Montagnani Marelli M, Sala A, Motta M, Limonta P (2004). Activation of the orphan nuclear receptor RORalpha counteracts the proliferative effect of fatty acids on prostate cancer cells: crucial role of 5-lipoxygenase. Int J Cancer.

[42] Nejati Moharrami N, Tande EB, Ryan L, Espevik T, Boyartchuk V. RORα controls inflammatory state of human macrophages. PLoS ONE. 2018;13:e0207374. 10.1371/journal.pone.0207374 PMC6261595 30485323

[43] Oh SK, Kim D, Kim K, Boo K, Yu YS, Kim IS (2019). RORα is crucial for attenuated inflammatory response to maintain intestinal homeostasis. Proc Natl Acad Sci USA.

[44] Delerive P, Monté D, Dubois G, Trottein F, Fruchart-Najib J, Mariani J (2001). The orphan nuclear receptor RORα is a negative regulator of the inflammatory response. EMBO Rep.

[45] Mao W, Xiong G, Wu Y, Wang C, Clair DS, Li JD, et al. RORα suppresses cancer-associated inflammation by repressing respiratory complex I-dependent ROS generation. Int J Mol Sci. 2021;22:10665. 10.3390/ijms221910665 PMC8509002 34639006

[46] Han S, Li Z, Han F, Jia Y, Qi L, Wu G (2019). ROR alpha protects against LPS-induced inflammation by down-regulating SIRT1/NF-kappa B pathway. Arch Biochem Biophys.

[47] Jiang Y, Zhou J, Zhao J, Hou D, Zhang H, Li L, et al. MiR-18a-downregulated RORA inhibits the proliferation and tumorigenesis of glioma using the TNF-α-mediated NF-κB signaling pathway. EBioMedicine. 2020;52:102651. 10.1016/j.ebiom.2020.102651 PMC7016377 32062354

