# Supplementary material for: Tumoral periprostatic adipose tissue exovesicles-derived miR-20a-5p regulates prostate cancer cell proliferation and inflammation through the RORA gene
Source: J Transl Med. 2024 Jul 15;22:661. doi: 10.1186/s12967-024-05458-3 (PMC11251289; doi:10.1186/s12967-024-05458-3)
Supplement: Supplementary file 1 — Supplementary Material 1 [file 12967_2024_5458_MOESM1_ESM.docx]

**Table S1.** Anthropometric and clinical characteristics (according to ISUP-GG criteria) of the pilot study patient’s:

| **Patient's Characteristics** | Median (Range) | N |
| --- | --- | --- |
| **Anthropometric parameters** |  |  |
| Age (years) | 67.00 (61.50, 71.75) | 4 |
| BMI (Kg/m^2^) | 24.35 (23.51, 25.50) | 4 |
| Prostatic volume (cc) | 41.25 (28.63, 110.50) | 4 |
| **Glycemic profile** |  |  |
| Glucose (mmol/L) | 5.17 (4.18, 5.81) | 4 |
| Insulin (pmol/L) | 53.96 (44.24, 107.23) | 4 |
| HOMA-IR | 1.82 (1.26, 3.74) | 4 |
| **Lipid profile** |  |  |
| Cholesterol Total (mmol/L) | 4.80 (5.58, 6.86) | 4 |
| HDL Cholesterol (mmol/L) | 1.18 (1.10, 1.45) | 4 |
| LDL Cholesterol (mmol/L) | 3.15 (2.39, 4.79) | 4 |
| Triglycerides (mmol/L) | 1.49 (1.09, 2.19) | 4 |
| **Hepatic profile** |  |  |
| AST (μkat/L) | 0.45 (0.28, 0.52) | 4 |
| ALT (μkat/L) | 0.36 (0.25, 0.45) | 4 |
| GGT (μkat/L) | 0.43 (0.31, 0.52) | 4 |
| **Renal profile** |  |  |
| Uric acid (μmol/L) | 379.56 (326.27, 412.79) | 4 |
| Urea (mmol/L) | 11.61 (6.70, 19.30) | 4 |
| Creatinine (μmol/L) | 91.50 (81.77, 95.25) | 4 |
| **Hormonal profile** |  |  |
| SHBG (nmol/l) | 48.70 (32.40, 56.80) | 4 |
| Testosterone (nmol/l) | 17.90 (11.68, 32.44) | 4 |
| **Tumoral markers** |  |  |
| Total PSA (ng/mL) | 12.74 (6.35, 18.90) | 4 |
| dPSA | 0.25 (0.16, 0.32) | 4 |

| **ISUP-GG** |  |  | **PPAT** | **PVAT** |
| --- | --- | --- | --- | --- |
| Low Risk | Group I | 0 | 0 | 0 |
|  | Group II | 0 | 0 | 0 |
| High Risk | Group III | 2 | 2 | 2 |
|  | Group IV | 2 | 2 | 2 |
|  | Group V | 0 | 0 | 0 |
| **T pathological stage** | ≤T2a | 1 | n=4 | n=4 |
|  | T3,T4 | 3 |  |  |
| **N pathological stage** | NX | 2 |  |  |
|  | N0 | 1 |  |  |
|  | N1 | 1 |  |  |

**Abbreviations**: BMI, body mass index; cc, centiliters; HOMA-IR, homeostatic model assessment for insulin resistance; HDL, high-density lipoprotein; LDL, low-density lipoprotein; AST, aspartate aminotransferase; ALT, alanine aminotransferase; GGT, gamma glutamyltransferase; SHBG, sex hormone-binding globulin; PSA, prostate specific antigen; dPSA, PSA density; PPAT, periprostatic adipose tissue; PVAT, perivesical adipose tissue; ISUP-GG, International Society of Urological Pathology Gleason Grade groups based on the Gleason score as follows: (Gleason score ≤ 6 - Group I; 3 + 4 = 7 - Group II; 4 + 3 = 7 - Group III; 4 + 4 = 8 - Group IV; and 9-10-Group V); T stage, Tumor category; N stage, Node category.

**Table S2.** Anthropometric and clinical characteristics (according to ISUP-GG criteria) of patients used in the validation study.

|  | **ISUP GG Classification** | |  |
| --- | --- | --- | --- |
| **Patient's Stratification** | **Low Risk** | **High Risk** |  |
|  | **(Group I and II)** | **(Group III, IV and V)** |  |
|  | **N=14** | **N=11** |  |
|  | **Median (Range)** | **Median (Range)** | ***p*-Value** |
| **Anthropometric parameters** |  |  |  |
| Age (years) | 66.00 (58.50, 71.25) | 64.50 (60.00, 68.50) | 0.814 |
| BMI (kg/m^2^) | 25.46 (24.31, 30.62) | 28.98 (25.94, 29.56) | 0.319 |
| Prostatic volume (cc) | 45.00 (26.50, 67.50) | 46.50 (35.00, 66.50) | 0.598 |
| **Glycaemic profile** |  |  |  |
| Glucose (mmol/L) | 5.61 (4.66, 6.70) | 5.05 (4.80, 5.97) | 0.504 |
| Insulin (pmol/L) | 84.10 (65.63, 102.54) | 62.09 (49.10, 125.40) | 0.650 |
| HOMA-IR | 3.27 (2.05, 3.77) | 1.82 (1.38, 3.78) | 0.414 |
| **Lipid profile** |  |  |  |
| Cholesterol (mmol/L) | 5.38 (4.63, 5.54) | 6.14 (4.54, 6.44) | 0.290 |
| HDL cholesterol (mmol/L) | 1.37 (1.14, 1.50) | 1.40 (1.13, 1.72) | 0.751 |
| LDL cholesterol (mmol/L) | 3.05 (2.57, 3.86) | 4.13 (2.68, 4.53) | 0.260 |
| Triglycerides (mmol/L) | 1.06 (0.90, 2.46) | 1.25 (1.06, 1.82) | 0.396 |
| **Hepatic profile** |  |  |  |
| AST (µkat/L) | 0.40 (0.32, 0.52) | 0.36 (0.30, 0.42) | 0.216 |
| ALT (µkat/L) | 0.43 (0.35, 0.80) | 0.38 (0.32, 0.48) | 0.217 |
| GGT (µkat/L) | 0.45 (0.32, 1.30) | 0.48 (0.33, 0.90) | 0.916 |
| **Renal profile** |  |  |  |
| Uric acid (µmol/L) | 309.32 (267.10, 382.00) | 365.53 (343.25, 405.12) | 0.139 |
| Urea (mmol/L) | 6.33 (4.91, 11.40) | 7.08 (6.10, 10.81) | 0.396 |
| Creatinine (μmol/L) | 79.65 (68.95, 89.73) | 86.63 (72.93, 94.81) | 0.203 |
| **Hormonal profile** |  |  |  |
| SHBG (nmol/L) | 56.80 (35.60, 60.40) | 40.50 (37.75, 50.73) | 0.347 |
| Testosterone (nmol/L) | 15.42 (11.39, 21.60) | 16.45 (13.84, 21.59) | 0.623 |
| **Tumoral marker** |  |  |  |
| Total PSA (μg/L) | 6.59 (4.85, 7.64) | 8.13 (5.72, 10.23) | 0.198 |
| dPSA | 0.18 (0.08, 0.26) | 0.16 (0.13, 0.19) | 0.709 |

| **ISUP-GG** |  | **N** | **PPAT** | **PVAT** |
| --- | --- | --- | --- | --- |
| **Low Risk** | Group I | 4 | 4 | 4 |
|  | Group II | 10 | 10 | 5 |
| **High Risk** | Group III | 8 | 8 | 5 |
|  | Group IV | 2 | 2 | 2 |
|  | Group V | 1 | 0 | 1 |
| **T pathological stage** | ≤T2a | 15 |  |  |
|  | T3,T4 | 10 |  |  |
| **N pathological stage** | NX | 19 |  |  |
|  | N0 | 4 |  |  |
|  | N1 | 2 |  |  |

**Abbreviations**: BMI, body mass index; cc, centiliters; HOMA-IR, homeostatic model assessment for insulin resistance; HDL, high-density lipoprotein; LDL, low-density lipoprotein; AST, aspartate aminotransferase; ALT, alanine aminotransferase; GGT, gamma glutamyltransferase; SHBG, sex hormone-binding globulin; PSA, prostate-specific antigen; dPSA, PSA density; PPAT, periprostatic adipose tissue; PVAT, perivesical adipose tissue; ISUP-GG, International Society of Urological Pathology Gleason Grade groups based on the Gleason score as follows: (Gleason score ≤ 6 - Group I; 3 + 4 = 7 - Group II; 4 + 3 = 7 - Group III; 4 + 4 = 8 - Group IV; and 9-10-Group V); T stage, Tumor category; N stage, Node category.

**Table S3.** Anthropometric and clinical characteristics of the CancerMIRNome patient’s database

| **Patient's Characteristics** |  |  |
| --- | --- | --- |
| **Anthropometric parameters** | Median (Range) | N |
| Age (years) | 60.29 (43.00, 72.00) | 52 |
|  |  |  |
| **ISUP-GG** |  |  |
| Low Risk | Group I | 5 |
|  | Group II | 25 |
| High Risk | Group III | 15 |
|  | Group IV | 3 |
|  | Group V | 4 |
| **T pathological stage** | ≤T2a | 29 |
|  | T3,T4 | 23 |
| **N pathological stage** | NX | 5 |
|  | N0 | 46 |
|  | N1 | 1 |

**Abbreviations:** ISUP-GG, International Society of Urological Pathology Gleason Grade groups based on the Gleason score as follows: (Gleason score ≤ 6 - Group I; 3 + 4 = 7 - Group II; 4 + 3 = 7 - Group III; 4 + 4 = 8 - Group IV; and 9-10-Group V); T stage, Tumor category; N stage, Node category.

**Table S4.** Anthropometric and clinical characteristics (according to ISUP-GG criteria) of patients used in the paraffin study.

|  | **ISUP GG Classification** | | |  |
| --- | --- | --- | --- | --- |
| **Patient's Stratification** | **Low Risk** | | **High Risk** |  |
|  | **(Group I and II)** | | **(Group III, IV and V)** |  |
|  | **N=18** | | **N=14** |  |
|  | **Median (Range)** | | **Median (Range)** | ***p*-Value** |
| **Anthropometric parameters** | |  |  |  |
| Age (years) | 67.5 (62.72, 69.75) | | 67.00 (63.50, 70.50) | 0.877 |
| BMI (kg/m^2^) | 27.03 (25.50, 28.85) | | 27.34 (25.77, 29.65) | 1.000 |
| Prostatic volume (cc) | 54.00 (29.75, 80.25) | | 46.00 (38.25, 60.50) | 0.644 |
| **Glycaemic profile** |  | |  |  |
| Glucose (mmol/L) | 5.75 (4.83, 6.87) | | 5.80 (4.96, 6.12) | 0.777 |
| Insulin (pmol/L) | 63.48 (50.60, 75.80) | | 75.08 (63.74, 99.11) | 0.080 |
| HOMA-IR | 2.23 (1.74, 3.43) | | 2.78 (2.12, 3.86) | 0.237 |
| **Lipid profile** |  | |  |  |
| Cholesterol (mmol/L) | 5.09 (4.40, 5.39) | | 5.03 (4.49, 5.79) | 0.681 |
| HDL cholesterol (mmol/L) | 1.53 (1.18, 1.67) | | 1.49 (1.23, 1.69) | 0.520 |
| LDL cholesterol (mmol/L) | 3.09 (2.51, 3.34) | | 3.03 (2.50, 3.58) | 0.959 |
| Triglycerides (mmol/L) | 1.05 (0.73, 1.73) | | 1.17 (0.89, 1.53) | 0.589 |
| **Tumoral marker** |  | |  |  |
| Total PSA (μg/L) | 5.60 (4.68, 7.08) | | 6.59 (5.36, 10.86) | 0.341 |
| dPSA | 0.16 (0.09, 0.19) | | 0.16 (0.10, 0.26) | 0.446 |

| **ISUP-GG** |  | N |
| --- | --- | --- |
| **Low Risk** | Group I | 18 |
|  | Group II | 0 |
| **High Risk** | Group III | 6 |
|  | Group IV | 6 |
|  | Group V | 2 |
| **T pathological stage** | ≤T2a | 32 |
|  | T3, T4 | 0 |
| **N pathological stage** | NX | 21 |
|  | N0 | 11 |
|  | N1 | 0 |

**Abbreviations**: BMI, body mass index; cc, centiliters; HOMA-IR, homeostatic model assessment for insulin resistance; HDL, high-density lipoprotein; LDL, low-density lipoprotein; PSA, prostate specific antigen; dPSA, PSA density; ISUP-GG, International Society of Urological Pathology Gleason Grade groups based on the Gleason score as follows: (Gleason score ≤ 6 - Group I; 3 + 4 = 7 - Group II; 4 + 3 = 7 - Group III; 4 + 4 = 8 - Group IV; and 9-10-Group V); T stage, Tumor category; N stage, Node category.
