# Supplementary material for: Tumoral periprostatic adipose tissue exovesicles-derived miR-20a-5p regulates prostate cancer cell proliferation and inflammation through the RORA gene
Source: J Transl Med. 2024 Jul 15;22:661. doi: 10.1186/s12967-024-05458-3 (PMC11251289; doi:10.1186/s12967-024-05458-3)
Supplement: Supplementary file 2 — Supplementary Material 2 [file 12967_2024_5458_MOESM2_ESM.pdf]

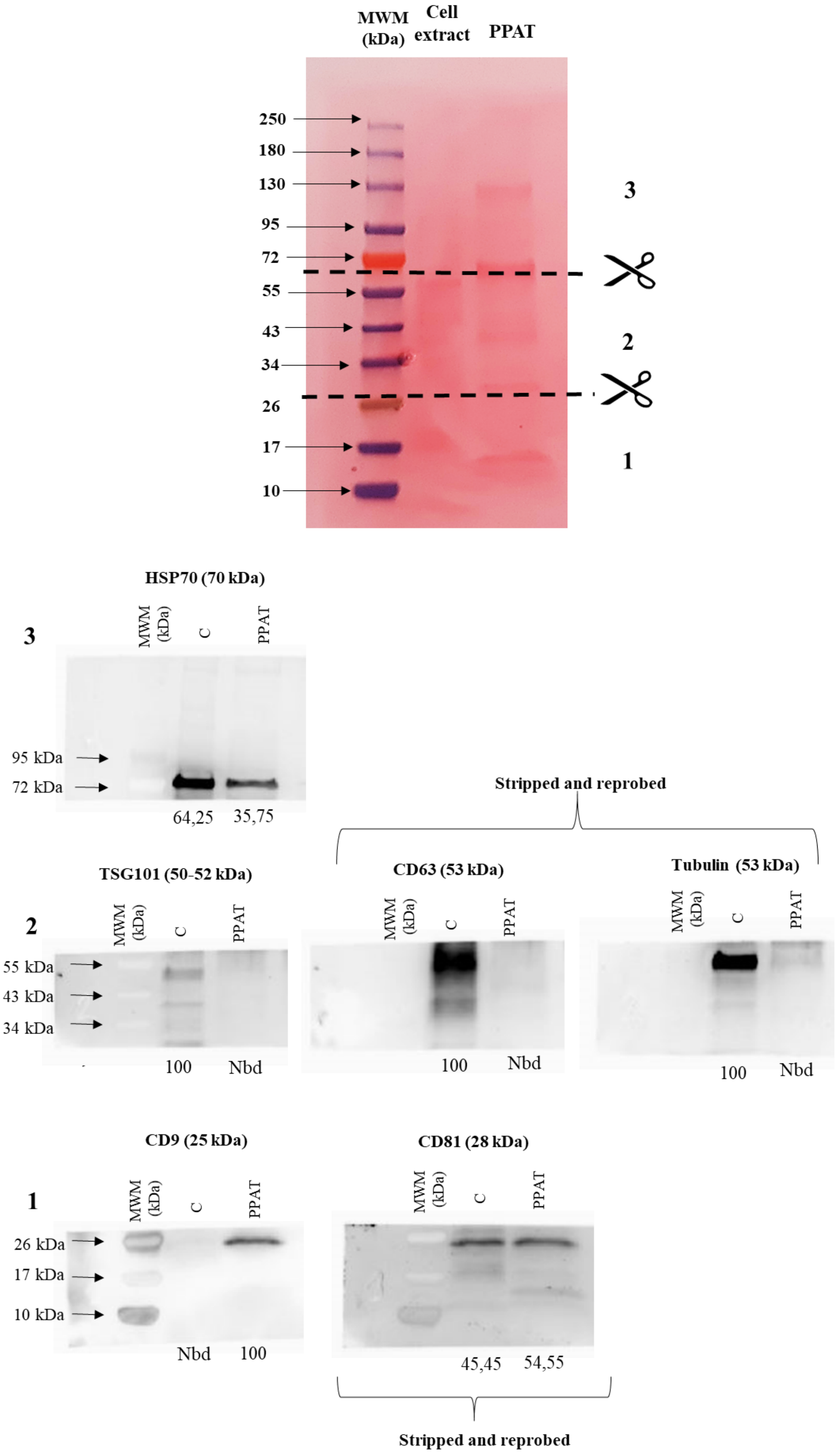
MWM (kDa)
Cell extract
PPAT
250
180
130
95
72
55
43
34
26
17
10
3
2
1
HSP70 (70 kDa)
MWM (kDa)
C
PPAT
95 kDa
72 kDa
64,25
35,75
Stripped and reprobed
TSG101 (50-52 kDa)
55 kDa
43 kDa
34 kDa
100
Nbd
CD63 (53 kDa)
100
Nbd
Tubulin (53 kDa)
100
Nbd
CD9 (25 kDa)
26 kDa
17 kDa
10 kDa
Nbd
100
CD81 (28 kDa)
45,45
54,55
Stripped and reprobed

Additional File 2: Figure S1. Complete Western Blot results referring to **Figure 1**. Ponceau stained images of gel-transferred membranes cut before antibody incubation to allow detection of CD9, TSG101, HSP70 proteins (1,2,3). Then stripping off was performed and membranes were reproved with CD63, CD81, and Tubulin antibodies (1 and 2). The numbers below the membranes represent the percentage of intensity.

Legend: C: Cell extract, Nbd: no band detected.
