# Supplementary material for: Tumoral periprostatic adipose tissue exovesicles-derived miR-20a-5p regulates prostate cancer cell proliferation and inflammation through the RORA gene
Source: J Transl Med. 2024 Jul 15;22:661. doi: 10.1186/s12967-024-05458-3 (PMC11251289; doi:10.1186/s12967-024-05458-3)
Supplement: Supplementary file 4 — Supplementary Material 4 [file 12967_2024_5458_MOESM4_ESM.pdf]

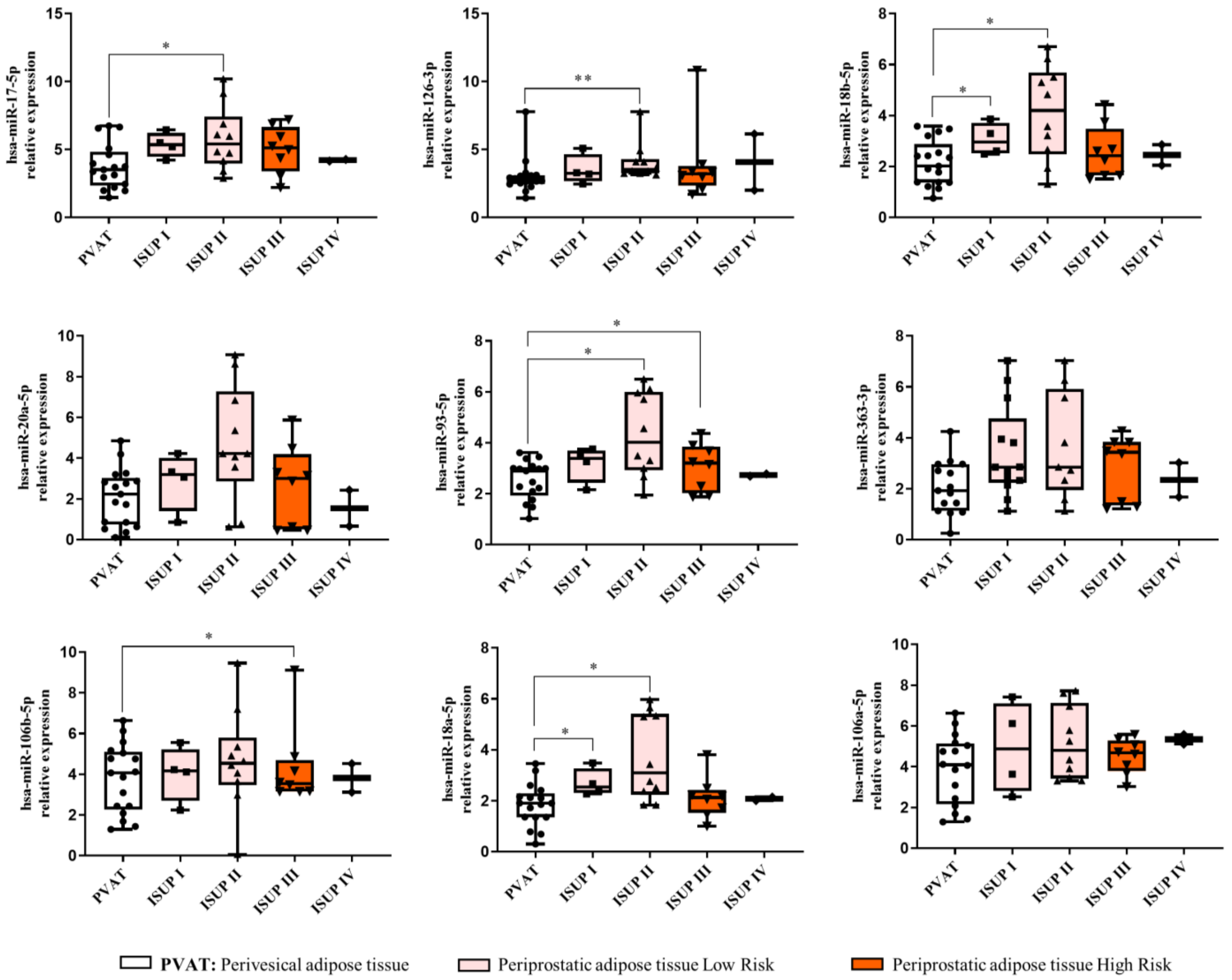


**Additional File 4: Figure S2.** Representation of the 9 discriminatory miRNAs in PPAT-EVs compared to PVAT-EVs segregated according to ISUP. Box plots showing median, quartiles, and extreme values of relative expression of discriminatory miRNAs. Symbols: * indicates significant differences, *$p$-*value*<0.05; **$p$-*value*<0.001.
