# Supplementary material for: Tumoral periprostatic adipose tissue exovesicles-derived miR-20a-5p regulates prostate cancer cell proliferation and inflammation through the RORA gene
Source: J Transl Med. 2024 Jul 15;22:661. doi: 10.1186/s12967-024-05458-3 (PMC11251289; doi:10.1186/s12967-024-05458-3)
Supplement: Supplementary file 5 — Supplementary Material 5 [file 12967_2024_5458_MOESM5_ESM.pdf]

FUNCTIONS	PROTEINS
**Cancer transformation and progression**<br>Regulation of RORA activity that attenuates the canonical Wnt pathway involved in cancer transformation and progression	**WNT5A**<br>**Wnt Family Member 5A**
**Cell growth**<br>Activation of transcriptional programs associated with oncogene and proto-oncogene mediated growth induction, tumor suppressor mediated growth arrest and replicative senescence, apoptosis and DNA repair	**KAT5**<br>**Lysine Acetyltransferase 5**
Mediation cellular responses to interleukins and other growth factors	**STAT3**<br>**Signal Transducer And Activator Of Transcription 3**
**Differentiation, inflammation and apoptosis**<br>Repression the transcriptional expression of genes genes involved in differentiation, inflammation, apoptosis etc..	**BCL-6**<br>**Transcription Repressor**
**T cell differentiation**<br>Transciption factor that mediates the differentiation of different T-cell type	**BATF**<br>**Basic Leucine Zipper ATF-Like Transcription Factor**
**Hypoxia, angiogenesis and tumor metastasis**<br>Transcriptional regulator of the adaptive response to hypoxia (involved in angiogenesis and tumor metastasis)	**HIF1A**<br>**Hypoxia-inducible factor 1-alpha**
**Circadian cycle**<br>ARTNL (CLOCK/NPAS2) forms BMAL1 heterodimer that transactivates the CRY and PER genes that are upregulated during the day but degraded at night (via phosphorylation and polyubiquitination). NR1D1 represses ARTNL transcription while RORA activates it. BAML1:CLOCK and BMAL1:NPAS2 heterodimers activate a set of genes that have E-box elements in their promoters (this confers circadian expression to the genes)	**ARNTL**<br>**Aryl Hydrocarbon Receptor Nuclear Translocator Like**<br>**NPAS2**<br>**Neuronal PAS Domain Protein 2**<br>**CLOCK**<br>**Clock Circadian Regulator**
RORA co-activator. Positive regulator of cicardian cycles	**NRIP1**<br>**Nuclear Receptor Interacting Protein 1**

Additional File 5: Figure S3. Table summarizing the function of the putative proteins interacting with genes resulting from the RORA STRING analysis (the colours of the protein name correspond to its corresponding node site at the STRING network in **Figure 3**).
