# Supplementary material for: Tumoral periprostatic adipose tissue exovesicles-derived miR-20a-5p regulates prostate cancer cell proliferation and inflammation through the RORA gene
Source: J Transl Med. 2024 Jul 15;22:661. doi: 10.1186/s12967-024-05458-3 (PMC11251289; doi:10.1186/s12967-024-05458-3)
Supplement: Supplementary file 6 — Supplementary Material 6 [file 12967_2024_5458_MOESM6_ESM.pdf]

RORA variant 1

miRNA	Site Position	LogitProb
hsa-miR-18b-5p	2473-2499	0,822
hsa-miR-93-5p	2463-2486	0,805
hsa-miR-17-5p	2463-2486	0,800
hsa-miR-106a-5p	2463-2486	0,800
hsa-miR-20a-5p	2463-2486	0,779
hsa-miR-106b-5p	2464-2486	0,779
hsa-miR-18a-5p	2464-2499	0,756
hsa-miR-17-5p	1893-1905	0,670
hsa-miR-106a-5p	1893-1905	0,670
hsa-miR-20a-5p	1893-1906	0,648
hsa-miR-20a-5p	1836-1847	0,616
hsa-miR-106b-5p	1897-1906	0,560
hsa-miR-93-5p	1863-1905	0,490
hsa-miR-126-3p	2574-2587	0,439
hsa-miR-93-5p	3718-3744	0,304
hsa-miR-17-5p	3718-3744	0,263
hsa-miR-20a-5p	3718-3744	0,263
hsa-miR-106a-5p	3718-3744	0,263
hsa-miR-106b-5p	3726-3744	0,253

RORA variant 2

miRNA	Site Position	LogitProb
hsa-miR-18b-5p	2473-2499	0,821
hsa-miR-93-5p	2463-2486	0,802
hsa-miR-106a-5p	2463-2486	0,799
hsa-miR-17-5p	2463-2486	0.799
hsa-miR-106b-5p	2464-2486	0,778
hsa-miR-20a-5p	2463-2486	0,778
hsa-miR-18a-5p	2464-2499	0,753
hsa-miR-106a-5p	1893-1905	0,663
hsa-miR-17-5p	1893-1905	0,663
hsa-miR-20a-5p	1893-1906	0,642
hsa-miR-20a-5p	1836-1847	0,638
hsa-miR-106b-5p	1897-1906	0,555
hsa-miR-93-5p	1863-1905	0,493
hsa-miR-126-3p	2574-2587	0,441
hsa-miR-93-5p	3718-3744	0,300
hsa-miR-106a-5p	3718-3744	0,261
hsa-miR-20a-5p	3718-3744	0,261
hsa-miR-17-5p	3718-3744	0,261
hsa-miR-106b-5p	3726-3744	0,251

RORA variant 3

miRNA	Site Position	LogitProb
hsa-miR-18b-5p	2449-2475	0,822
hsa-miR-93-5p	2439-2462	0,802
hsa-miR-17-5p	2439-2462	0,798
hsa-miR-106a-5p	2439-2462	0,798
hsa-miR-20a-5p	2439-2462	0,777
hsa-miR-106b-5p	2440-2462	0,775
hsa-miR-18a-5p	2440-2475	0,750
hsa-miR-17-5p	1869-1881	0,672
hsa-miR-106a-5p	1869-1881	0,672
hsa-miR-20a-5p	1869-1882	0,650
hsa-miR-20a-5p	1812-1823	0,618
hsa-miR-106b-5p	1873-1882	0,560
hsa-miR-93-5p	1839-1881	0,489
hsa-miR-126-3p	2550-2563	0,440
hsa-miR-93-5p	3694-3720	0,304
hsa-miR-17-5p	3694-3720	0,261
hsa-miR-20a-5p	3694-3720	0,261
hsa-miR-106a-5p	3694-3720	0,261
hsa-miR-106b-5p	3702-3720	0,250

RORA variant 4

miRNA	Site Position	LogitProb
hsa-miR-18b-5p	2162-2188	0,824
hsa-miR-93-5p	2152-2175	0,805
hsa-miR-17-5p	2152-2175	0,798
hsa-miR-106a-5p	2152-2175	0,798
hsa-miR-20a-5p	2152-2175	0,777
hsa-miR-106b-5p	2153-2175	0,776
hsa-miR-18a-5p	2153-2188	0,753
hsa-miR-20a-5p	1525-1536	0,696
hsa-miR-17-5p	1582-1594	0,676
hsa-miR-106a-5p	1582-1594	0,676
hsa-miR-20a-5p	1582-1595	0,656
hsa-miR-106b-5p	1586-1595	0,564
hsa-miR-93-5p	1552-1594	0,444
hsa-miR-126-3p	2263-2276	0,443
hsa-miR-93-5p	3407-3433	0,312
hsa-miR-17-5p	3407-3433	0,272
hsa-miR-20a-5p	3407-3433	0,272
hsa-miR-106a-5p	3407-3433	0,272
hsa-miR-106b-5p	3415-3433	0,262

Additional File 6: Figure S4. Tables of all logistic probabilities of binding between miRNAs and each *RORA* variant are shown with the site position.
