# Supplementary material for: Tumoral periprostatic adipose tissue exovesicles-derived miR-20a-5p regulates prostate cancer cell proliferation and inflammation through the RORA gene
Source: J Transl Med. 2024 Jul 15;22:661. doi: 10.1186/s12967-024-05458-3 (PMC11251289; doi:10.1186/s12967-024-05458-3)
Supplement: Supplementary file 7 — Supplementary Material 7 [file 12967_2024_5458_MOESM7_ESM.pdf]

A

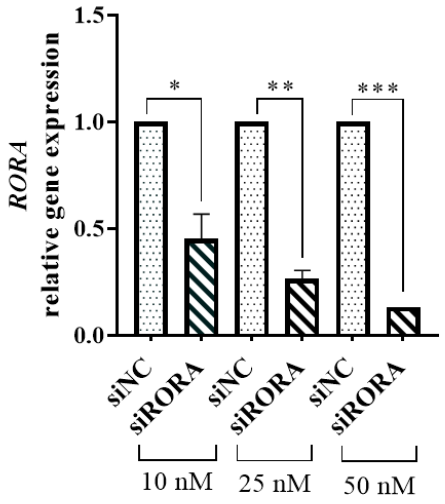

B

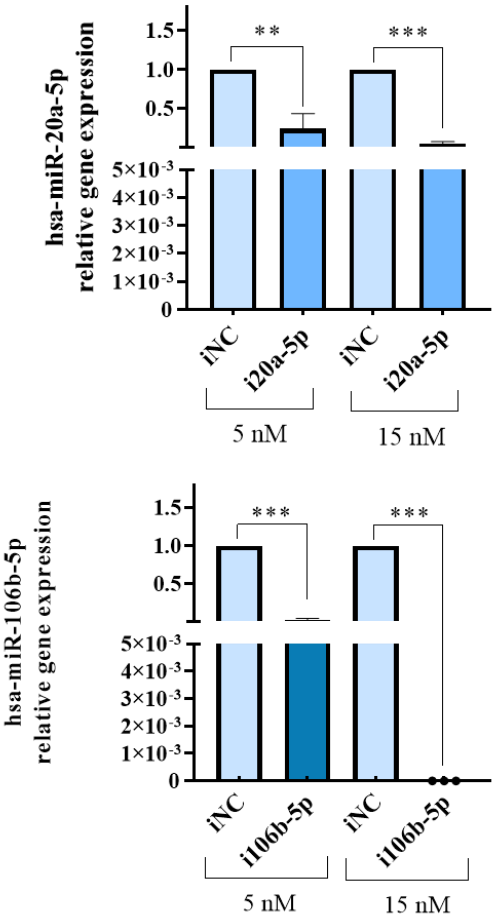

Additional File 7: Figure S5. **A** Dose response of siRORA in 22Rv1 cells at 24 hours: 10, 25, and 50 nM. Results are presented as relative expressions with mean and SEM of triplicate experiments. **B** Graph shows hsa-miR-20a-5p and hsa-miR-106b-5p expression after treating 22Rv1 cells with 5 and 15 nM of their respective inhibitors for 24 hours. Symbols: * indicates significant differences, **p-value*<0.05; ** *pvalue*<0.01; ****p-value*<0.001.
