# Supplementary material for: Tumoral periprostatic adipose tissue exovesicles-derived miR-20a-5p regulates prostate cancer cell proliferation and inflammation through the RORA gene
Source: J Transl Med. 2024 Jul 15;22:661. doi: 10.1186/s12967-024-05458-3 (PMC11251289; doi:10.1186/s12967-024-05458-3)
Supplement: Supplementary file 8 — Supplementary Material 8 [file 12967_2024_5458_MOESM8_ESM.pdf]

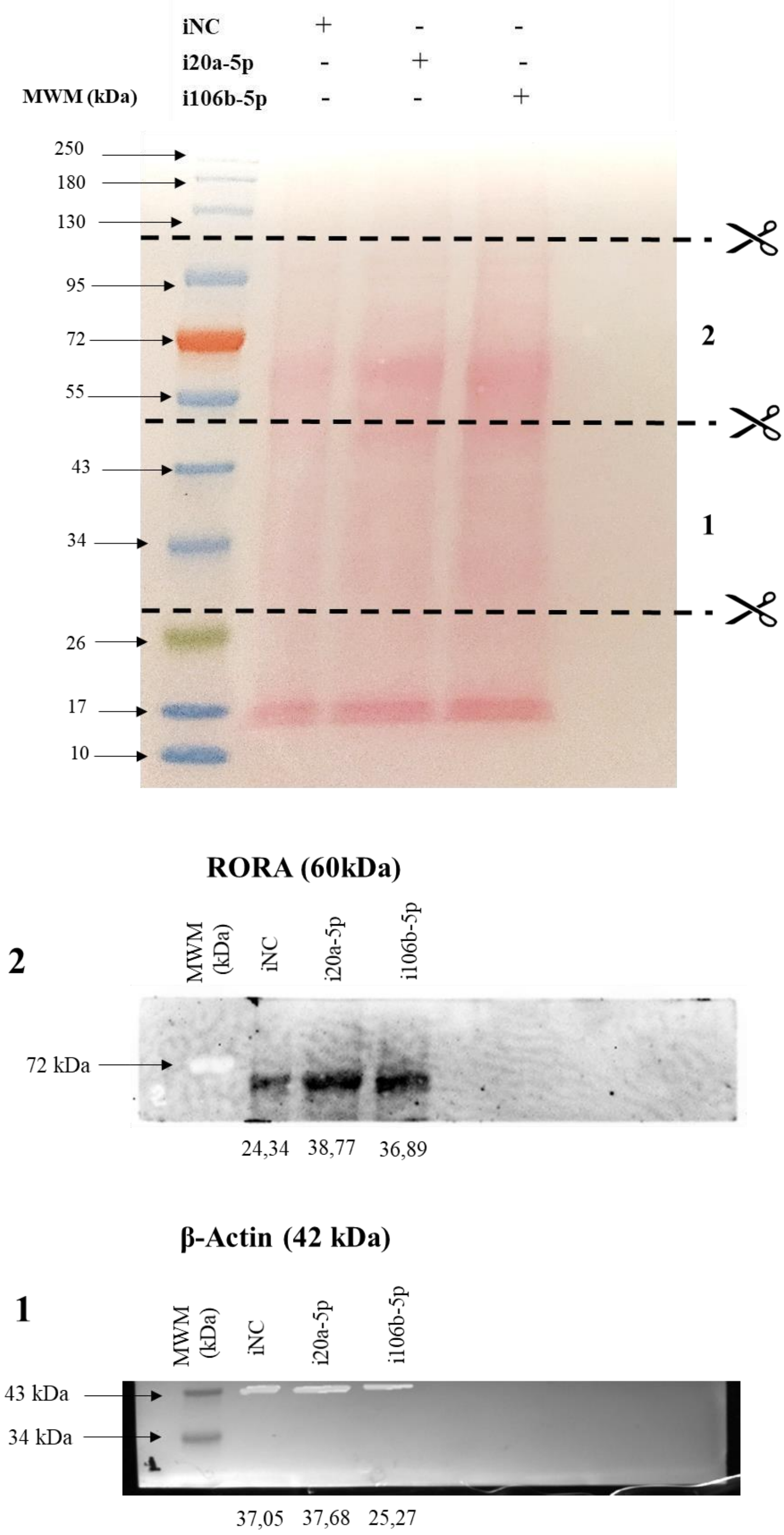

Additional File 8: Figure S6. Complete Western blot result referring to **Figure 5D**. Ponceau stained images of gel transferred membranes cut before antibody incubation to allow detection of RORA and βActin proteins. The numbers below membranes represent the percentage of intensity.
