# Supplementary material for: Tumoral periprostatic adipose tissue exovesicles-derived miR-20a-5p regulates prostate cancer cell proliferation and inflammation through the RORA gene
Source: J Transl Med. 2024 Jul 15;22:661. doi: 10.1186/s12967-024-05458-3 (PMC11251289; doi:10.1186/s12967-024-05458-3)
Supplement: Supplementary file 9 — Supplementary Material 9 [file 12967_2024_5458_MOESM9_ESM.pdf]

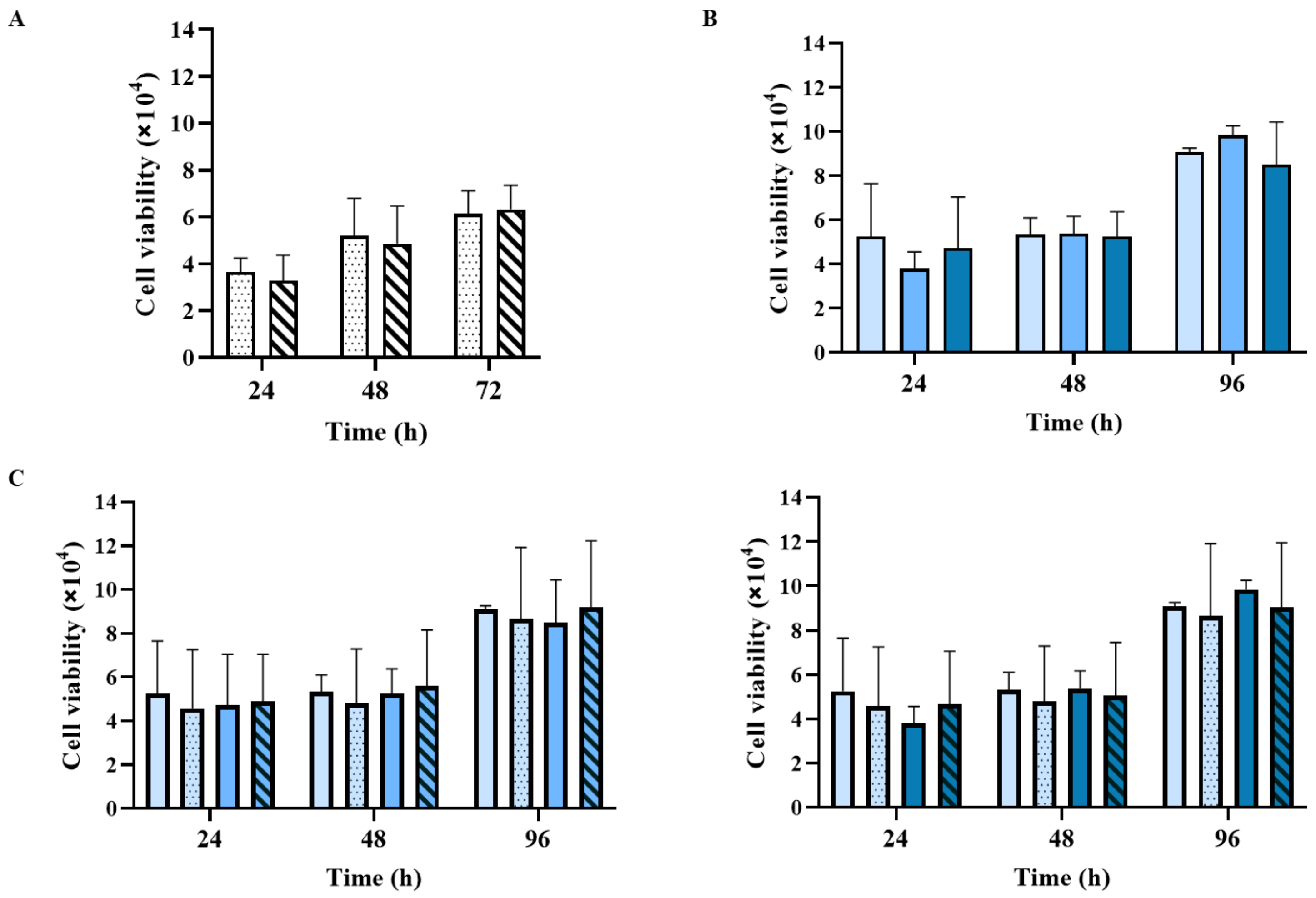

Additional File 9: Figure S7. Cell Proliferation assays of 22Rv1 transfected with siRORA. **A** miR-20a-5p inhibitor (i20-1-5p) or miR-106b-5p inhibitor (i106b-5p). **B** or with the combination of each miRNA with siRNA RORA. **C** at different time points.
